# Magnetic–Dielectric Synergy in One-Dimensional Metal Heterostructures for Enhanced Low-Frequency Microwave Absorption

**DOI:** 10.1007/s40820-025-01995-8

**Published:** 2026-01-05

**Authors:** Feiyue Hu, Peigen Zhang, Pei Ding, Shuo Zhang, Bingbing Fan, Ali Saffar Shamshirgar, Wei Zheng, Wenwen Sun, Longzhu Cai, Haijiao Xie, Qiyue Shao, Johanna Rosen, ZhengMing Sun

**Affiliations:** 1 State Key Laboratory of Engineering Materials for Major Infrastructure, School of Materials Science and Engineering, Southeast University, Nanjing, 211189 People’s Republic of China; 2 School of Materials Science and Engineering, Zhengzhou University, Zhengzhou, 450001 People’s Republic of China; 3 Department of Engineering, Faculty of Environment, Science and Economy, University of Exeter, Exeter, EX4 4QF UK; 4 Materials Design Division, Department of Physics, Chemistry and Biology (IFM), Linköping University, 581 83 Linköping, Sweden; 5 State Key Laboratory of Millimeter Waves, School of Information Science and Engineering, Southeast University, Nanjing, 210096 People’s Republic of China; 6 Hangzhou Yanqu Information Technology Co., Ltd, Hangzhou, 310003 People’s Republic of China

**Keywords:** Low frequency microwave absorption, Magnetic–dielectric synergy, MAX phase, CoNi@SnO_2_@Sn heterostructure, Thermal conductivity

## Abstract

Microwave absorption (MA) materials often face poor synergy between impedance matching and attenuation in the low-frequency range. Balancing permittivity and permeability through magnetic–dielectric synergy is a promising strategy to address this issue. To realize the synergy, herein, Sn whiskers with an in situ oxide layer served as substrates for magnetic-loss-active CoNi nanosheet growth, forming a hierarchical CoNi@SnO_2_@Sn (CNS) heterostructure. The CNS absorber achieves a minimum reflection loss (*RL*_min_) value of − 62.29 dB with an effective absorption bandwidth (EAB) of 2.2 GHz, covering the entire C-band with 70% absorption at only 2.61 mm thickness. The nanosheet design of CoNi enhances magnetic anisotropy to promote natural resonance, while the conductive Sn core and abundant Sn/SnO_2_ and CoNi/SnO_2_ heterointerfaces facilitate conduction loss and dielectric polarization. When composited into a thermoplastic polyurethane (TPU) matrix, the resulting CNS/TPU-2 film (20 wt% CNS) exhibits an RL_min_ value of -61.04 dB and a 2.5 GHz EAB. Its in-plane and through-plane thermal conductivities reach 2.41 and 0.51 W m^−1^ K^−1^, representing 4.1 and 2.6 times those of pure TPU films, respectively, facilitating heat dissipation from protected devices. This work provides valuable insights into magnetic–dielectric synergy for low-frequency MA of 1D metal-based materials, offering promising potential for 5G communications and flexible electronics.

**Supplementary Information:**

The online version contains supplementary material available at 10.1007/s40820-025-01995-8.

## Highlights

- The hierarchical structure of CoNi nanosheets wrapped on one-dimensional Sn whiskers enhances magnetic anisotropy and dielectric losses, enabling strong magnetic–dielectric synergy.
- The CoNi@SnO_2_@Sn (CNS) filler achieves − 62.29 dB reflection loss and 2.2 GHz bandwidth, fully covering the C-band with > 70% absorption, outperforming most low-frequency absorbers.
- A flexible CNS/TPU film exhibits superior low-frequency microwave absorption and thermal conductivity, expanding its potential applications in communication electronics.

**Supplementary Information:**

The online version contains supplementary material available at 10.1007/s40820-025-01995-8.

## Graphical abstract

**Figure:**
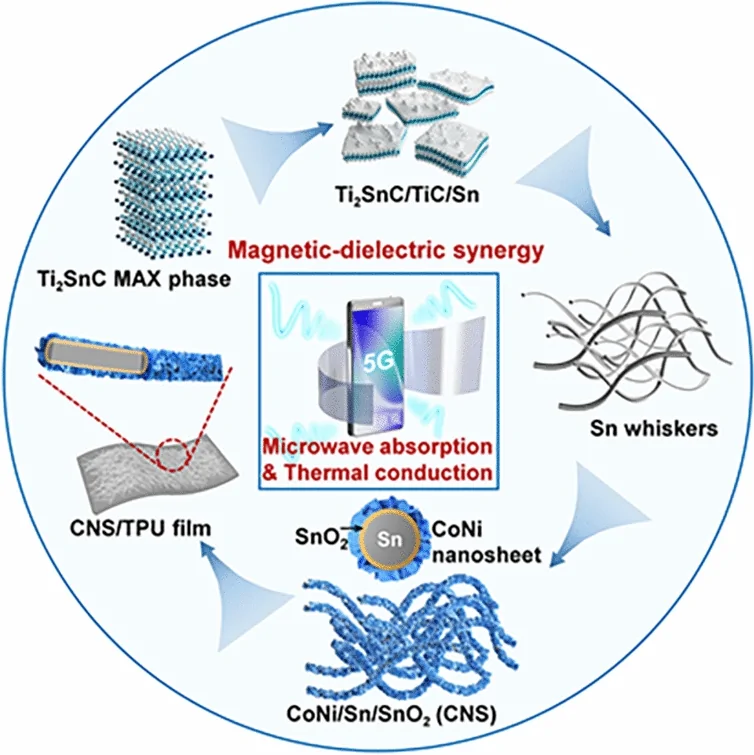

## Introduction

The rapid advancement of 5G communication technology has significantly accelerated the development of mobile internet [1, 2]. However, it has also intensified electromagnetic interference (EMI) and radiation pollution within 5G-specific frequency bands, particularly in the S-band (2–4 GHz), C-band (4–8 GHz), and sub-bands such as n77 (3.3–4.2 GHz), n78 (3.3–3.8 GHz), and n79 (4.4–5.0 GHz), resulting in signal disruption and equipment malfunction [3–5]. Microwave absorption (MA) materials are capable of attenuating incident electromagnetic waves through mechanisms such as dielectric and magnetic losses, offering effective solutions to EMI and radiation-related issues [6, 7]. Nevertheless, most reported MA materials demonstrate optimal performance in the mid- to high-frequency ranges, primarily in the X-band (8–12 GHz) and Ku-band (12–18 GHz) [8]. For the lower-frequency S- and C-bands, current MA materials often suffer from narrow effective absorption bandwidth (EAB) and require relatively large matching thicknesses [9]. These limitations are mainly ascribed to the weakened synergy between impedance matching and attenuation capability at low frequencies, which is strongly governed by the intrinsic dielectric constant and magnetic permeability of the material [10].

Magnetic metals such as Fe, Co, Ni, and their alloys, which possess high magnetic permeability and moderate dielectric properties, are commonly utilized for low-frequency MA applications [11]. Their unfilled 3d orbitals facilitate the formation of external magnetic moments, enabling microwave attenuation via mechanisms such as eddy current loss and natural resonance. However, the classical Snoek limit imposes a trade-off between magnetic permeability and resonance frequency, restricting these materials’ performance at the higher end of the low-frequency range [12]. Moreover, magnetic loss typically decreases sharply with increasing frequency, and the natural resonance range is relatively narrow. In contrast, dielectric-type absorbers exhibit better stability in practical applications, and their low-frequency absorption performance relies on a high dielectric constant and electrical conductivity [13]. Nonetheless, excessive conductivity in dielectric loss-based absorbers tends to cause strong reflection of microwaves, leading to impedance mismatch and severely narrowing the EAB in the low-frequency regime. To enhance low-frequency MA performance, two main strategies have been pursued. One approach involves engineering ferromagnetic materials into one-dimensional (1D) or flake-like morphologies to increase shape anisotropy, thereby strengthening natural resonance and helping to circumvent the trade-off imposed by the Snoek limit [14]. Enhanced magnetic anisotropy helps maintain high magnetic permeability while elevating the resonance frequency, thereby improving low-frequency absorption. For instance, Li et al. [15] synthesized anisotropic flake-like FeNi alloys via mechanical ball milling, achieving a minimum reflection loss (*RL*_min_) value of − 20.0 dB and a low-frequency EAB of 2.1 GHz at a thickness of 3.3 mm. Wu et al. reported a hierarchical magnetic microchain with a core–shell structure of two-dimensional Fe_*x*_Co_1−*x*_OOH nanosheets vertically anchored on the surface of one-dimensional Co microchains. Such long-range anisotropic magnetic microchains achieve a RL below − 40 dB at a matching thickness of 4.5 mm in the C-band [16]. Although these strategies improve low-frequency MA performance to some extent, their limited attenuation capability still constrains both absorption efficiency and the EAB. The second strategy focuses on magnetic–dielectric synergistic design, which aims to enhance the magnetic loss capability of dielectric materials, balance dielectric constant and magnetic permeability, and optimize impedance matching while introducing diverse attenuation mechanisms [17]. For example, Yang et al. [18] fabricated a composite absorber by combining a FeCoNi medium-entropy alloy with a 1D carbon-based framework (FeCoNi/CF) via an electrospinning technique. The resulting absorber exhibited an EAB of 1.3 GHz at a thickness of 2 mm in the low-frequency region. However, both the absorption efficiency and bandwidth remain insufficient for practical application.

Consequently, a central challenge in current research lies in achieving simultaneous improvement in RL_min_, EAB, and absorber thickness by optimizing the component composition and structural architecture to enhance low-frequency impedance matching and wave attenuation capability. Additionally, the energy dissipated by MA materials is typically converted into heat, while electronic devices also generate waste heat during operation [19]. The accumulation of heat can raise the device temperature, degrade performance, and even cause failure. Therefore, high thermal conductivity is critical for MA materials in low-frequency applications. 1D metal materials have emerged as promising candidates for low-frequency MA applications owing to their high electrical conductivity and ease of network formation [20]. These materials can offer tunable dielectric or magnetic loss characteristics and facilitate efficient thermal conduction pathways. For instance, He et al. [21] synthesized chain-like CoNi via a solvothermal route using polyvinylpyrrolidone (PVP) as a directing agent and incorporated it into a polydimethylsiloxane (PDMS) matrix to fabricate flexible CoNi/PDMS films. The composite film containing 18 vol% CoNi achieved the best low-frequency MA performance, with an RL_min_ value of -56.7 dB, an EAB of 1.04 GHz at 4.1 mm thickness, and in-plane and through-plane thermal conductivities of 2.05 and 0.61 W m^−1^ K^−1^, respectively. These values were significantly higher than those of films using spherical CoNi or pure PDMS. However, microwave loss and thermal conductivity of chain-like CoNi are still constrained by grain boundaries, falling short of the performance achievable with single-crystalline metallic whiskers or nanowires [22, 23]. Therefore, ideal 1D metal-based low-frequency MA materials should feature single-crystalline structures and high aspect ratios, which facilitate the formation of efficient conductive and thermal pathways—characteristics exemplified by metal whiskers.

Despite advances in the development of metal whiskers, their application remains limited due to challenges such as restricted composition, low production efficiency, and impedance mismatch with microwaves [24]. Most studies have focused on noble metal whiskers such as Au and Ag [25]. Conventional synthesis methods, including physical vapor deposition (PVD) and chemical vapor deposition (CVD), require sophisticated equipment and high energy consumption, limiting scalability [26, 27]. Solvothermal methods pose safety and environmental concerns [28]. In recent years, the spontaneous growth of metal whiskers has attracted attention as a promising route for 1D metal fabrication [29]. With the assistance of templating strategies, this approach could potentially broaden the material composition and enhance fabrication efficiency. For example, Kosinova et al. [30] deposited a 10 nm Au atomic layer on glass substrates using electron beam evaporation and observed the spontaneous formation of single-crystalline Au whiskers driven by capillary diffusion. Similarly, Tohmyoh et al. [31] reported Ag whisker formation in a related system. More recently, Kimura et al. [32] achieved large-scale growth of Al whisker arrays at designated locations by controlling atomic diffusion within solid films. Despite these advances, the processes still rely on ultrathin metal films and are limited by high costs and restricted elemental diversity. In this context, spontaneous whisker growth from A-site elements in ternary transition metal carbides and nitrides (MAX phases) has drawn increasing attention [33]. MAX phases are a class of layered transition metal carbides or nitrides (M_n+1_AX_n_, where M = transition metal, A = group element, X = C or N) [34] featuring a hexagonally close-packed structure, in which M and A layers alternate and X atoms occupy octahedral sites. This phenomenon, combined with chemical environment modulation of the A-site and subsequent mechanochemical decomposition, offers a novel approach for metal whisker fabrication [35, 36]. Compared to template-based and solvothermal methods, this strategy provides significant advantages in terms of equipment cost, production efficiency, and elemental diversity [37]. Moreover, to address impedance mismatch in high-dielectric materials, interfacial engineering, particularly the construction of core–shell architectures, has proven to be an effective strategy for optimizing impedance matching and enhancing polarization loss [38]. Within various heterostructures, metal/metal oxide interfaces provide an effective approach to tailoring dielectric properties and enhancing electromagnetic wave dissipation, which is well aligned with the compositional characteristics of low-frequency microwave-absorbing materials such as magnetic metals, their alloys, and ferrites.

Based on these considerations, this study proposes an interface-engineered 1D metal-based strategy for low-frequency MA application. Single-crystalline Sn whiskers were first synthesized by optimizing the mechanochemical decomposition process of the Ti_2_SnC MAX phase. These whiskers exhibited an in situ surface oxide layer (SnO_2_) with high dielectric loss potential, but impedance mismatch limited their EAB to only 0.9 GHz. To overcome this, a hydrothermal method was employed to coat nanosheets-like CoNi on the SnO_2_ surface, forming a 1D hierarchical heterostructure denoted as CoNi@SnO_2_@Sn (CNS). Both theoretical calculations and experimental analyses were conducted to investigate the low-frequency absorption performance and mechanisms of this heterostructure. The formation of two metal/semiconductor interfaces (Sn/SnO_2_ and CoNi/SnO_2_) with differing work functions generated built-in electric fields, while abundant lattice defects induced by CoNi alloying enhanced dielectric polarization loss at low frequencies. Additionally, the CoNi nanosheets grown on the SnO_2_ layer enhanced magnetic anisotropy and elevated the frequency of natural resonance. The resulting macroscopic magnetic coupling further improved dissipation of low-frequency microwaves. Ultimately, the synergistic magnetic–dielectric effect endowed the 1D metal-based absorber with excellent low-frequency MA performance. When integrated into flexible composite films, these materials demonstrated broadband absorption and high thermal conductivity, offering significant potential for applications in 5G communication and advanced electronic devices.

## Experimental Section

### Raw Materials

The raw materials required for this process are all analytical reagents and can be used without further purification. Ti (99.0%, 250 mesh), Sn (99.5%, 200 mesh), graphite (99.9%, 500 mesh), CoCl_2_·6H_2_O (99%), NiCl_2_·6H_2_O (99%), and N,N-dimethylacetamide (DMAC, 99.0%) and were all purchased from Shanghai Macklin Biochemical Co., Ltd (Shanghai, China). Thermoplastic polyurethane (TPU, 99%, 1185 A) was purchased from BASF SE (Germany), and hydrazine hydrate (N_2_H_4_·H_2_O, 80%) was obtained from Shanghai Lingfeng Chemical Reagent Co., Ltd (Shanghai, China).

### Synthesis of Sn Whiskers

First, Ti, Sn, and graphite powders were mixed in a 2:1:1 molar ratio for 24 h and then heated at 1330 °C for 2 h in argon to obtain the Ti_2_SnC MAX phase. Afterward, the as-synthesized Ti_2_SnC was ball-milled at 650 r min^−1^ for 8 h (ball-to-powder ratio of 10:1), during which the jar was opened several times to replenish air. The ball-milled Ti_2_SnC powders were placed in air at room temperature for several hours to grow Sn whiskers, resulting in a flocculent mixture (Sn/TiC(Ti_2_SnC)). The pure Sn whiskers can be separated and purified in two ways, either by picking up the upper flocs of the mixture with tweezers or by collecting the flocs by washing the mixture in deionized water with multiple ultrasonic washes.

### Synthesis of CoNi@SnO_2_@Sn (CNS) and CNS/TPU Films

A total of 100 mg of Sn whiskers, 1 mmol of CoCl_2_·6H_2_O, and 1 mmol of NiCl_2_·6H_2_O were added to 50 mL of deionized water and stirred magnetically for 30 min. Subsequently, 5 mL of N_2_H_4_·H_2_O was added dropwise, followed by continued stirring for 20 min. The resulting mixture was then transferred into a Teflon-lined stainless-steel autoclave and subjected to hydrothermal treatment at 160 °C for 15 h in an oven. After the reaction, the product was collected by centrifugation, washed thoroughly, and vacuum-dried at 50 °C for 6 h to yield the CoNi@SnO_2_@Sn (CNS, also denoted as CNS-2) composite. Following the same procedure, when 2 mmol of CoCl_2_·6H_2_O was used in place of both CoCl_2_·6H_2_O and NiCl_2_·6H_2_O, the resulting product was denoted as Co@Sn (CS). Similarly, replacing the two precursors with 2 mmol of NiCl_2_·6H_2_O yielded Ni@Sn (NS); using 0.5 mmol of each precursor gave rise to CNS-1; and using 1.5 mmol of each gave rise to CNS-3. In addition, when Sn whiskers were excluded and only 1 mmol of CoCl_2_·6H_2_O and 1 mmol of NiCl_2_·6H_2_O were used, the product was designated as CoNi.

Subsequently, 1 g of TPU and 9 mL of DMAC were added into a glass vial, sealed, and subjected to shaking on a three-dimensional shaker for 12 h to obtain a homogeneous TPU/DMAC solution. An appropriate amount of the solution was then transferred to a new glass vial using a pipette, followed by the addition of the CNS sample. The mixture was thoroughly shaken to ensure uniform dispersion. The resulting mixture was poured into a glass petri dish and dried in a vacuum oven at 50 °C for 8 h to form a film. A series of composite films, denoted as CNS/TPU-1, CNS/TPU-2, CNS/TPU-3, and CNS/TPU-4, were prepared by varying the mass ratio of CNS to TPU (1:9, 2:8, 3:7, and 4:6, respectively).

### Characterization

X-ray diffractometer (XRD, Bruker D8-Discover, Cu K_α_ radiation, working at 40 kV) was used to identify the phase composition. A scanning electron microscope (SEM, ZEISS Crossbeam 350) was used to observe the morphology. High-resolution transmission electron microscopy (HRTEM) and selected area electron diffraction (SAED) were also conducted on a FEI Tecnai 2100 transmission electron microscope (TEM) equipped with an energy-dispersive spectrometer (EDS) to characterize the samples. The JEM-2100F TEM, including off-axis electron holography was conducted to further study the microstructure of the samples. X-ray photoelectron spectroscopy (XPS, Thermo Scientific K-Alpha, ESCALAB 250XI) was used to study the chemical state of the sample surface. Magnetic hysteresis loops were investigated by the LakeShore7404 vibrating sample magnetometer (VSM) with magnetic field strength of − 20,000 to 20,000 Oe at room temperature. Tensile strength and elongation at break of film samples were tested using an electronic universal testing machine (MTS, EXCEED Model E43). The thermal conductivity (*λ*) of the film samples was evaluated using the formula *λ* = *D*·*C*_p_·*ρ*, where the thermal diffusivity coefficient (*D*) was determined through the laser flash method conducted in an Ar atmosphere (LFA467, NERMANY). The specific heat (*C*_p_) of the samples was measured using differential scanning calorimetry (DSC8000). The density (*ρ*) was determined by assessing the dimensions and mass of the disk-shaped (diameter of 25 mm) samples. At least three measurements were taken for each set of samples and averaged to ensure accuracy. An infrared thermal camera was utilized to record the temperature and infrared images during heating and cooling.

The electromagnetic parameters (S-parameters, dielectric constant, and permeability) of ring-shaped samples (50 wt% loading in paraffin wax, outer diameter *ϕ*_o_ = 7.0 mm, inner diameter *ϕ*_i_ = 3.04 mm, thickness ≈ 2.00 mm) containing Sn whiskers and magnetically modified materials (CoNi, NS, CS, and CNS) were measured using a vector network analyzer (VNA, Agilent N5244A). Additionally, for film samples, the precursor solution was vacuum-dried in a ring mold, demolded to obtain coaxial ring structures, and tested using the same VNA.

## Results and Discussion

### Materials Characterization

**Fig. 1:**
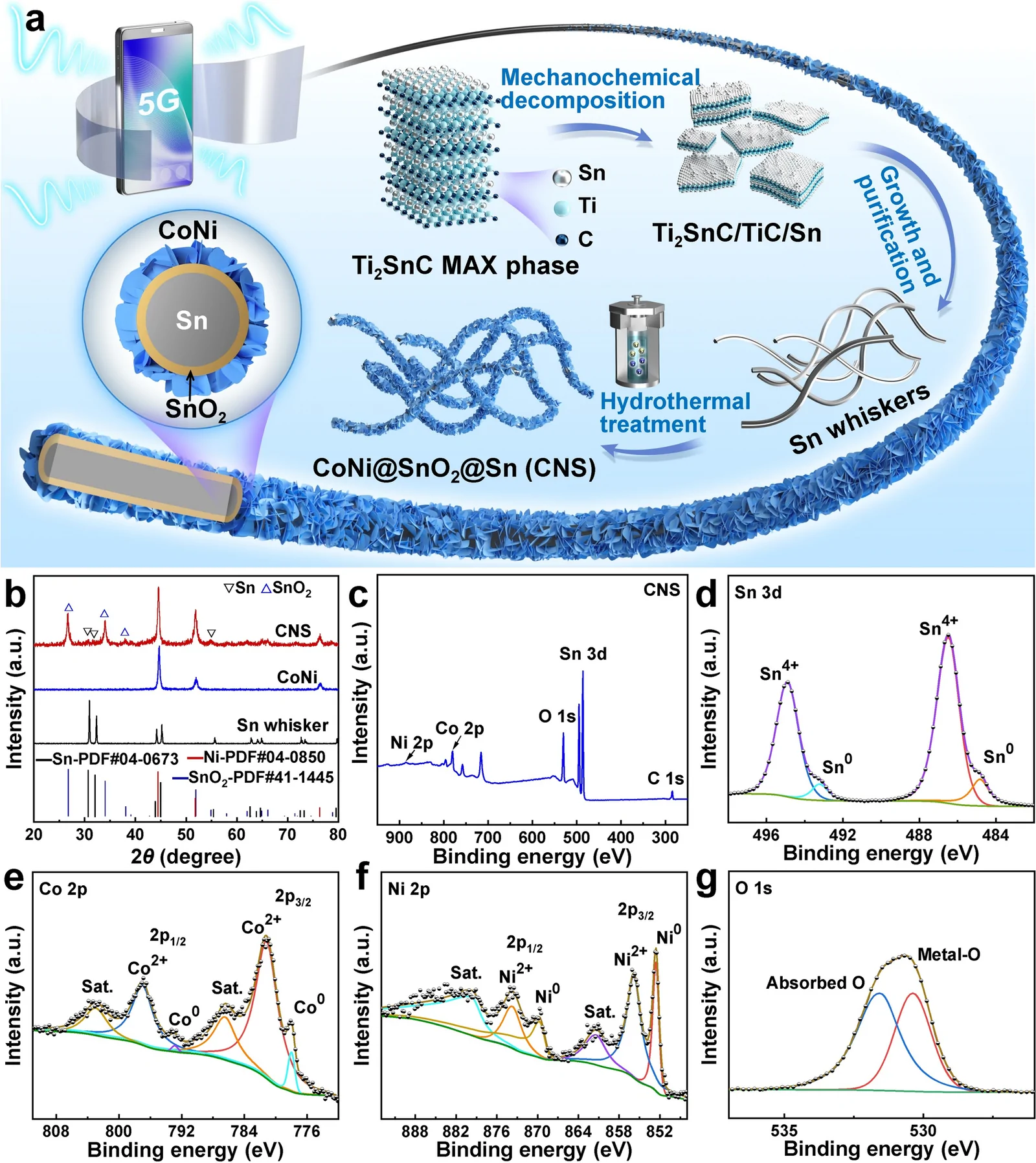
**a** Schematic of the preparation process of the CNS sample. **b** XRD patterns of Sn whisker, CoNi and CNS. **c** XPS survey, **d** Sn 3*d* spectrum, **e** Co 2*p* spectrum, **f** Ni 2*p* spectrum, and **g** O 1*s* spectrum of CNS

A schematic illustration of the synthesis of hierarchical 1D metal-based MA materials for low-frequency applications is shown in Fig. 1a. First, 1D Sn whiskers were synthesized as conductive cores via a modified mechanochemical decomposition method, utilizing the spontaneous growth behavior of A-site metals in MAX phases. Building on our previous work [39], we introduced O_2_ repeatedly during the decomposition of Ti_2_SnC MAX phase to increase the exposure of Sn layers, thereby promoting the release of high-energy active Sn atoms. These atoms spontaneously aggregate into Sn whiskers within hours, demonstrating very high efficiency in whisker formation. Due to their high aspect ratio, the whiskers can be easily separated from the matrix via stirring and sonication. As shown in Fig. S1, the Sn whiskers are single-crystalline and coated with an in situ SnO_2_ shell, providing both conductive and dielectric losses, consistent with our earlier findings [40, 41]. Using the Sn whiskers as templates, CoNi alloy nanosheets were grown on their surface via hydrothermal treatment, forming a hierarchical CoNi@SnO_2_@Sn (CNS) structure. Here, Sn serves as the conductive core, SnO_2_ as the intermediate layer, and CoNi as the outer shell. This design exploits interfacial polarization at the Sn/SnO_2_ and CoNi/SnO_2_ interfaces along with magnetic losses from the CoNi nanosheets to enhance low-frequency MA via magnetic–dielectric synergy. Figure 1b shows the XRD patterns. The Sn whiskers derived from mechanochemical decomposition exhibit β-Sn peaks (PDF#04-0673) [42], while the hydrothermally deposited CoNi matches the Ni card (PDF#04-0850) [43]. Due to the in situ oxidation of Sn under hydrothermal conditions, additional SnO_2_ peaks (PDF#41-1445) [44] are observed in the CNS sample, indicating that the oxide layer has transformed into a crystalline state. Meanwhile, the decrease in Sn peak intensity is attributed to the nucleation of Ni on the SnO₂ surface. XPS was used to investigate the surface chemical states of CNS (Fig. 1c–g). The survey spectrum (Fig. 1c) confirms the presence of Co, Ni, Sn, and O, with C 1*s* calibrated to 284.8 eV. In Fig. 1d, the Sn 3*d* spectrum shows peaks at 494.9 and 486.5 eV corresponding to Sn^4+^, and at 493.2 and 484.8 eV corresponding to Sn^0^, confirming the presence of a SnO_2_ layer on metallic Sn [45]. The Co 2*p* and Ni 2*p* spectra (Fig. 1e, f) show characteristic peaks at 792.9 and 778.0 eV (Co 2*p*_1/2_ and 2*p*_3/2_), 869.7 and 852.6 eV (Ni 2*p*_1/2_ and 2*p*_3/2_), confirming the formation of CoNi alloy on the whiskers [46]. The O 1*s* spectrum (Fig. 1g) shows peaks at 530.4 and 531.6 eV, corresponding to metal–oxygen bonds and adsorbed oxygen, respectively. These analyses confirm the successful synthesis of the 1D metal-based hierarchical structure. Specifically, the amorphous oxide layer on the Sn whisker surface transforms into a crystalline SnO_2_ shell during the hydrothermal process. Simultaneously, Co^2+^/Ni^2+^ ions in the solution are reduced and alloyed under reducing conditions, preferentially nucleating and growing on the SnO_2_ surface. The crystallization of SnO_2_ provides abundant grain boundaries, defects, and surface hydroxyl sites, significantly lowering the nucleation barrier and facilitating the in situ deposition of the CoNi alloy. As the reaction proceeds, stable metal/oxide interfaces gradually form between CoNi and SnO_2_ through Co–O/Ni–O coordination bonds and local lattice matching. This process not only stabilizes the CoNi/SnO_2_ heterointerface structurally but also provides additional active sites for charge accumulation and polarization, which is expected to enhance interfacial polarization loss and magnetic–dielectric coupling effects.

**Fig. 2:**
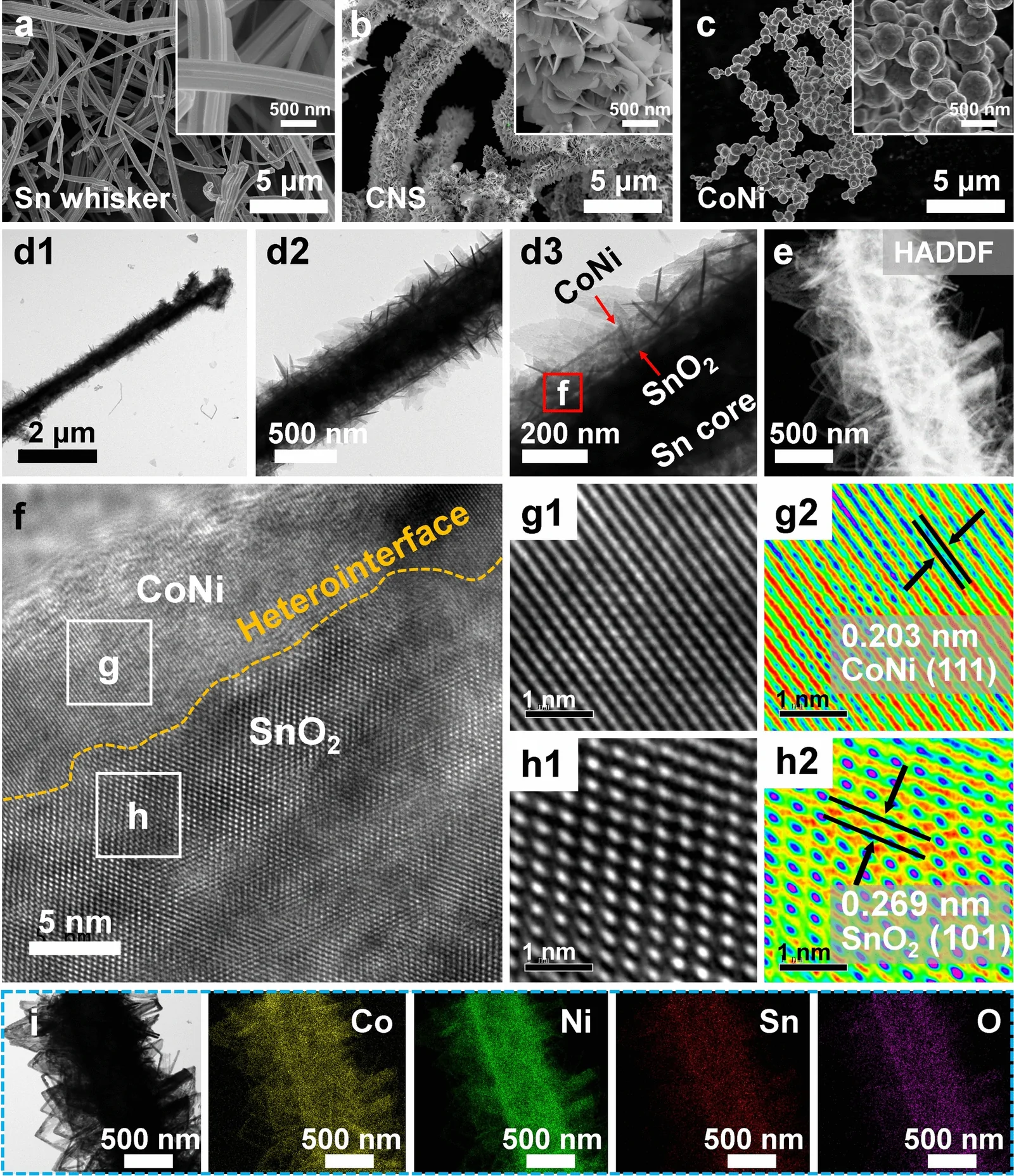
SEM images of **a** Sn whisker, **b** CNS, and **c** CoNi (enlarged image inset in the upper right corner). **d1**–**d3** TEM, **e** HADDF, **f**–**h** HRTEM images (**g1**, **g2** and **h1**, **h2** originate from the **g** and **h** regions, respectively), and **i** the corresponding EDS mapping of CNS

Figure 2a–c shows SEM images of the Sn whiskers, CNS, and CoNi chains, respectively. The Sn whiskers exhibit a fibrous morphology with diameters ranging from 100 to 1000 nm and lengths extending to tens or even hundreds of micrometers. After hydrothermal coating of the magnetic alloy, CoNi nanosheets are uniformly grown on the surface of the whiskers, forming numerous 2D/1D metal/semiconductor heterointerfaces, which are considered highly effective for dielectric loss-based microwave attenuation. In contrast, under the same hydrothermal conditions but without Sn whiskers as nucleation templates, pure CoNi forms chain-like structures composed of interconnected particles with an average diameter of ~ 350 nm. Figure S2 reveals that in the Ni-coated Sn whiskers (NS), the outer layer consists of granular Ni, whereas the Co-coated Sn whiskers (CS) are covered by nanosheet-like Co. Figure S3 shows that pure Ni forms chain-like structures with diameters of ~ 1.5 μm, while pure Co exists as irregular spherical particles with diameters of 2–3 μm, with a few nanosheets distributed on their surfaces. Furthermore, as shown in Fig. S4, increasing the CoNi loading induces a morphological transition in the outer layer of CNS from nanosheets to particles.

Figure 2d, e presents the TEM image and the corresponding high-angle annular dark field (HAADF) image of the CNS sample, respectively. These clearly confirm the heterostructure where Sn whiskers are coated with CoNi nanosheets, with distinguishable CoNi outer shells, SnO_2_ intermediate layers, and Sn cores. The HRTEM image in Fig. 2f displays a distinct CoNi/SnO_2_ heterointerface. On either side of this interface, lattice fringes with spacings of 0.203 and 0.269 nm are observed in Fig. 2g, h, corresponding to the (111) plane of CoNi and the (101) plane of SnO_2_, respectively [45, 46]. The energy-dispersive spectroscopy (EDS) mapping image in Fig. 2i reveals that Co and Ni elements are mainly distributed in the outermost layer, while Sn and O are concentrated in the inner regions, further verifying the successful synthesis of the hierarchical heterostructure.

### Low-Frequency MA Performance of CNS

**Fig. 3:**
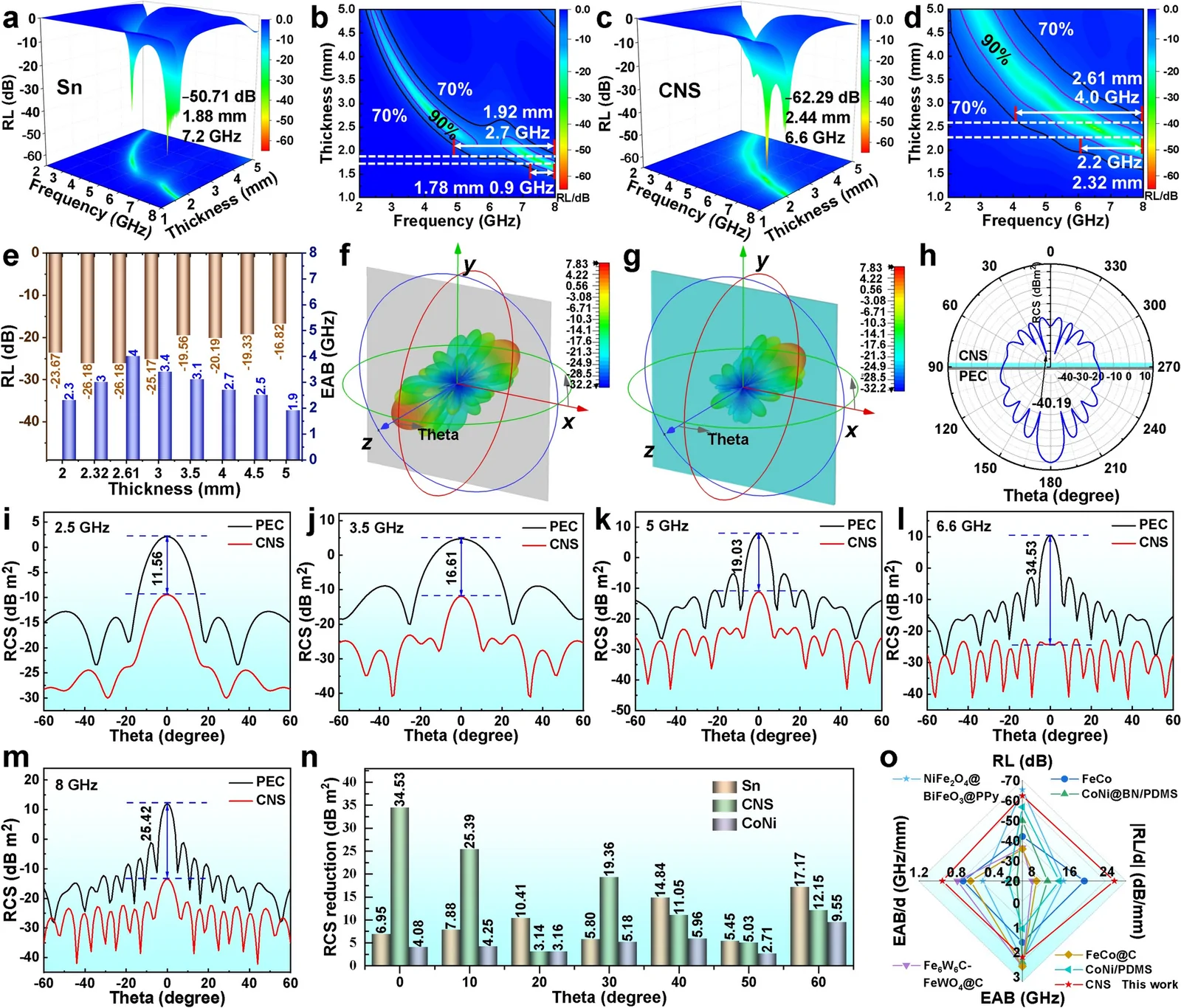
Low-frequency MA properties. The RL with different thicknesses of **a**, **b** Sn whisker and **c**, **d** CNS. **e** the RL value and EAB-70% of CNS. RCS plots for **f** PEC plate and **g**, **h** PEC plate covered with CNS at 6.6 GHz. 2D RCS plots of CNS-coated PEC plates at different low-frequency points for **i** 2.5 GHz, **j** 3.5 GHz, **k** 5 GHz, **l** 6.6 GHz, and **m** 8 GHz, respectively. **n** RCS reduction values of Sn. CoNi and CNS at 6.6 GHz. **o** Comparison of the performance of low-frequency microwave absorbers

The MA performance of all samples was evaluated using the coaxial-line method. Based on transmission line theory, the RL values of samples with different thicknesses were calculated using the following equation [47, 48]:

1 $$RL = 20\lg \left| {\frac{{Z_{{{\mathrm{in}}}} - Z_{0} }}{{Z_{{{\mathrm{in}}}} + Z_{0} }}} \right|,$$

2 $$Z_{{{\mathrm{in}}}} = Z_{0} \sqrt {\mu_{r} {/}\varepsilon_{r} } \tanh \left[ {j\left( {\frac{2\pi fd}{c}} \right)\sqrt {\mu_{r} \varepsilon_{r} } } \right],$$

where *Z*_in_ is the input impedance of the absorber, Z_0_ is the impedance of free space, d is the absorber thickness, f is the frequency, and c is the speed of light. Generally, an RL value below −10 dB (corresponding to over 90% absorption) is considered qualified for practical applications, and the frequency range where this is achieved is termed the EAB. However, owing to the intrinsic trade-off between impedance matching and attenuation in the low-frequency region (2–8 GHz), an RL threshold of − 4 dB (corresponding to ~ 60% absorption) is generally considered sufficient to satisfy practical application requirements [18]. In this work, we propose a high-standard low-frequency MA evaluation framework, which incorporates three metrics: 90% absorption (RL < − 10 dB, denoted EAB or EAB-90%), 70% absorption (RL < − 5.2 dB, denoted EAB-70%), and S-band 60% absorption (RL < − 4 dB, denoted EAB-60%). Figure 3a, b displays the 3D and 2D plots of the absorption performance of Sn whiskers as a function of thickness (1–5 mm) within 2–8 GHz. The Sn whiskers exhibit an RL_min_ value of − 50.71 dB, with an EAB-90% of 0.9 GHz and an EAB-70% of 2.7 GHz at matching thicknesses below 2 mm, indicating good low-frequency MA potential. Figure S5 shows that CoNi alloy chains yield an RL_min_ value of only − 16.12 dB at 5.00 mm thickness, with EAB-90% and EAB-70% of 1.5 GHz (at 5.00 mm) and 3.9 GHz (at 3.82 mm), respectively, demonstrating broadband low-frequency absorption capability.

Under the same conditions, CNS exhibits significantly enhanced MA properties across 2–8 GHz, with an RL_min_ value of − 62.29 dB at 6.6 GHz and a thickness of just 2.44 mm (Fig. 3c). Its EAB-90% and EAB-70% reach 2.2 GHz and 4.0 GHz (fully covering the C-band), respectively, with all matching thicknesses below 3 mm (Fig. 3d). Further evaluation over the extended range of 2–18 GHz (Fig. S6) shows that CNS achieves an EAB-90% of 5.4 GHz (at a thickness of only 1.26 mm), which is three times that of Sn whiskers, underscoring the advantage of the magnetic–dielectric synergistic strategy. As shown in Fig. S7, NS and CS samples exhibit inferior RL_min_ and EAB compared to CNS, indicating that alloying (CoNi) enhances low-frequency MA performance more effectively than single-metal coating. Additionally, we plotted the EAB-70% and RL_min_ values of CNS at various thicknesses (Fig. 3e) to demonstrate its broad bandwidth absorption behavior. Figures S8–S10 summarize the EAB-60% values in the S-band and EAB-70% values in the C-band for Sn, CoNi, NS, CS, and CNS. Notably, CNS achieves 95% absorption across the entire S-band (2.1–4.0 GHz) at a thickness of only 4.00 mm, indicating excellent low-frequency performance. As shown in Figs. S11 and S12, increasing the CoNi loading initially improves the CNS sample’s low-frequency MA performance, followed by a decline, suggesting an optimal loading exists.

Furthermore, simulations were conducted in CST Microwave Studio by applying the material as a low-frequency absorbing coating on a perfect electric conductor (PEC) plate to evaluate its stealth performance. In accordance with the metal-backed model of transmission line theory, this setup was used to simulate the radar cross section (RCS) of the coating under far-field conditions [49]. The RCS results at 6.6 GHz for bare PEC and PEC coated with Sn whiskers, CoNi, or CNS are shown in Figs. 3f–h and S13. The bare PEC plate exhibits the strongest scattering, while all coated samples reduce the radar wave scattering intensity. CNS-coated PEC shows the lowest RCS, with values below -15 dB m^2^ across -90° to 90° (Fig. 3h), satisfying the general stealth requirement (< − 10 dB m^2^). RCS reduction, defined as the difference in RCS between bare PEC and absorber-coated PEC, is an important stealth parameter. To further validate the broadband absorption performance, we simulated the RCS values and reductions at 2.5, 3.5, 5, 6.6, and 8 GHz. As shown in Figs. 3i–n and S14, CNS achieves over 10 dB m^2^ RCS reduction at all these frequencies. Notably, at 6.6 GHz, the CNS coating yields a maximum RCS reduction value of 34.53 dB m^2^ at normal incidence (*θ* = 0°), significantly surpassing other samples. Finally, to highlight the advantage of our magnetic–dielectric synergistic design, we benchmarked CNS against recently reported advanced low-frequency absorbers (e.g., 1D magnetic alloys and magnetic alloys coated with carbon) from top-tier journals (Fig. 3o and Table S1) [4, 9, 10, 14, 21, 50]. The results confirm that the fabricated 1D hierarchical CNS serves as an outstanding absorber with thin thickness, strong absorption, and wide bandwidth, making it a highly promising candidate for next-generation low-frequency electromagnetic protection applications.

### Low-Frequency MA Mechanism of CNS

**Fig. 4:**
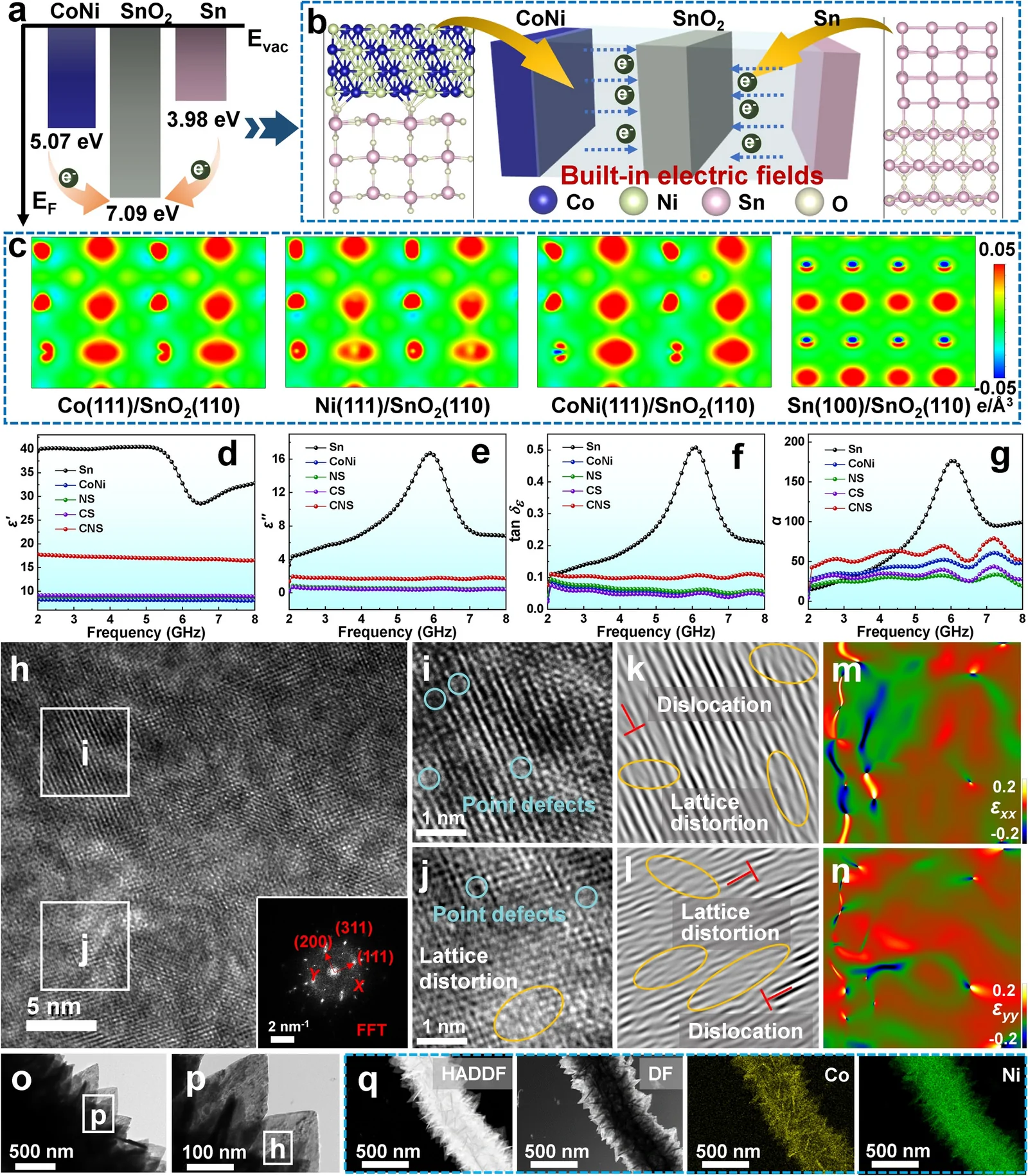
Low-frequency microwave absorption mechanisms: dielectric loss. **a** Scheme of electron transfer between CoNi/SnO_2_ and Sn/SnO_2_ heterointerfaces. **b** The DFT calculated configuration model and the formation of built-in electric field of these heterointerfaces. **c** Differential charge density mapping of the Co/SnO_2_, Ni/SnO_2_, CoNi/SnO_2_, and Sn/SnO_2_ heterointerfaces (the blue and red regions refer to electron depletion and accumulation, respectively). **d** *ε*′, **e** *ε*″, **f** tan δ_ε_, and **g** Attenuation constant (*α*) of Sn, CoNi, CS, NS, and CNS. **h** HRTEM image, with insets showing corresponding fast Fourier transform (FFT) patterns of the outer layer of the CNS. **i**, **j** Localized enlargements of the region in **h**. **k**–**l** The inverse fast Fourier transform (IFFT) filtered images of **i** and **j**, respectively. The strain distribution images along the *ε*_xx_ **m** and *ε*_yy_ **n** directions in region **h** (colors from green to dark blue indicating compressive strains and red to bright yellow indicating tensile strains). **o**, **p** TEM images, **q** HADDF, DF, and EDS mapping of CNS

To elucidate the enhanced low-frequency MA performance of CNS, we investigated its microwave loss mechanisms. First-principles calculations were performed to determine the work functions of each component (Fig. S15), revealing that Sn possesses the lowest work function (3.98 eV), the CoNi alloy lies between pure Co and Ni, and SnO_2_ exhibits the highest work function (7.09 eV), indicative of its semiconducting nature [44]. Based on these differences, various metal/semiconductor interfaces were constructed. Figure 4a, b shows the configuration models and the formation of built-in electric fields at these heterogeneous interfaces, where electrons escape from the metal surface and flow toward the semiconductor. Density functional theory (DFT) was used to calculate the differential charge distributions at different interfaces (Figs. 4c and S16), indicating that the charge separation in the CoNi/SnO_2_ interface is slightly greater than that in the Co/SnO_2_ and Ni/SnO_2_ interfaces, while Sn/SnO_2_ exhibits the highest degree of charge separation. Consequently, compared with pure Co or Ni, the CoNi alloy forms a stronger built-in electric field with SnO_2_, and Sn/SnO_2_ generates the strongest field. Under the influence of an external alternating electromagnetic field, the direction of these built-in fields periodically reverses, inducing orientation polarization (i.e., interfacial polarization), which dissipates electromagnetic energy [51].

Furthermore, the electromagnetic parameters of each sample were measured using a VNA (Fig. 4d–f). Due to its high conductivity, Sn whiskers exhibit the largest permittivity and loss tangent in the 2–8 GHz range, which can cause impedance mismatch and reflect incident waves. Upon the introduction of magnetic components, ε′, ε″, and tan δ_ε_ values are significantly reduced, and CNS exhibits higher dielectric loss capability compared to CS, NS, and CoNi. In addition, the attenuation constant α, a key indicator of electromagnetic energy dissipation, is calculated as follows [52]:

3 $$\alpha = \frac{\sqrt 2 \pi f}{c} \times \sqrt {\left( {\mu^{\prime \prime } \varepsilon ^{\prime\prime} - \mu^{\prime } \varepsilon^{\prime } } \right) + \sqrt {\left( {\mu^{\prime \prime } \varepsilon^{\prime \prime } - \mu^{\prime } \varepsilon^{\prime } } \right)^{2} + \left( {\mu^{\prime } \varepsilon^{\prime \prime } + \mu^{\prime \prime } \varepsilon^{\prime } } \right)^{2} } } .$$

As shown in Fig. 4g, CNS demonstrates the highest α value within 2–4.5 GHz, while Sn whiskers dominate in the 4.5–8 GHz range, followed by CNS, indicating the excellent attenuation capacity of the 1D hierarchical structure enabled by interface engineering.

The aforementioned DFT calculations and electromagnetic analyses suggest that alloyed structures enhance dielectric loss. Therefore, further HRTEM characterization of the alloy nanosheets was conducted. As illustrated in Fig. 4h–l, randomly selected CoNi nanosheets contain abundant point defects. Inverse fast Fourier transform (IFFT) filtered images reveal numerous edge dislocations (indicated by the symbol ‘⊥’) and pronounced lattice distortions. Geometric phase analysis (GPA) of the HRTEM images (Fig. 4m, n) reveals the microstrain distribution within the CoNi nanosheets, indicating that tensile strain is concentrated in several regions. This phenomenon arises from local structural defects caused by the random occupation of Co and Ni atoms with different atomic radii, which induces localized strain fields. In addition, during the hydrothermal process, the SnO_2_ shell transforms from an amorphous to a crystalline state, leading to lattice mismatch and stress concentration at the CoNi/SnO_2_ interface. These combined effects give rise to abundant defects within the CoNi regions. These defects disrupt lattice periodicity and serve as charge-trapping centers, causing nonuniform local electric fields. When subjected to external electric fields, the separation of positive and negative charge centers leads to displacement polarization (i.e., defect-induced dipolar polarization), resulting in the dissipation of electromagnetic energy. Figure 4o–q presents the TEM, HADDF, and EDS mapping of the CNS sample, validating the accuracy of selecting the CoNi nanosheet region for analysis.

To further clarify the dielectric loss mechanism of CNS, ε″-ε′ (Cole–Cole) plots were generated (Fig. S17). According to Debye relaxation theory, the relationship between ε′ and ε″ can be described by the following equation [53]:

4 $$\left( {\varepsilon^{\prime } - \frac{{\varepsilon_{s} - \varepsilon_{\infty } }}{2}} \right)^{2} + \left( {\varepsilon^{\prime \prime } } \right)^{2} = \left( {\frac{{\varepsilon_{s} - \varepsilon_{\infty } }}{2}} \right)^{2} ,$$

where *ε*_s_ and *ε*_∞_ are the static and infinite-frequency permittivity, respectively, *τ* is the relaxation time, and *ω* (*ω* = 2*πf*) is the angular frequency. As described in Eq. (4), a semicircle in the *ε*′–*ε*″ plot indicates a Debye relaxation process, with each semicircle corresponding to a distinct relaxation. The Cole–Cole plots of the as-prepared 1D metal-based composites show distorted semicircles, followed by linear segments in the high-frequency region, attributable to pronounced conduction losses. Comparing the number of Cole–Cole semicircles across all samples reveals that CNS exhibits the highest number of relaxation processes. This is attributed to the interfacial polarization caused by the built-in electric fields at the multicomponent heterogeneous interfaces and the defect-induced dipolar polarization from the CoNi alloy nanosheets, as discussed above.

**Fig. 5:**
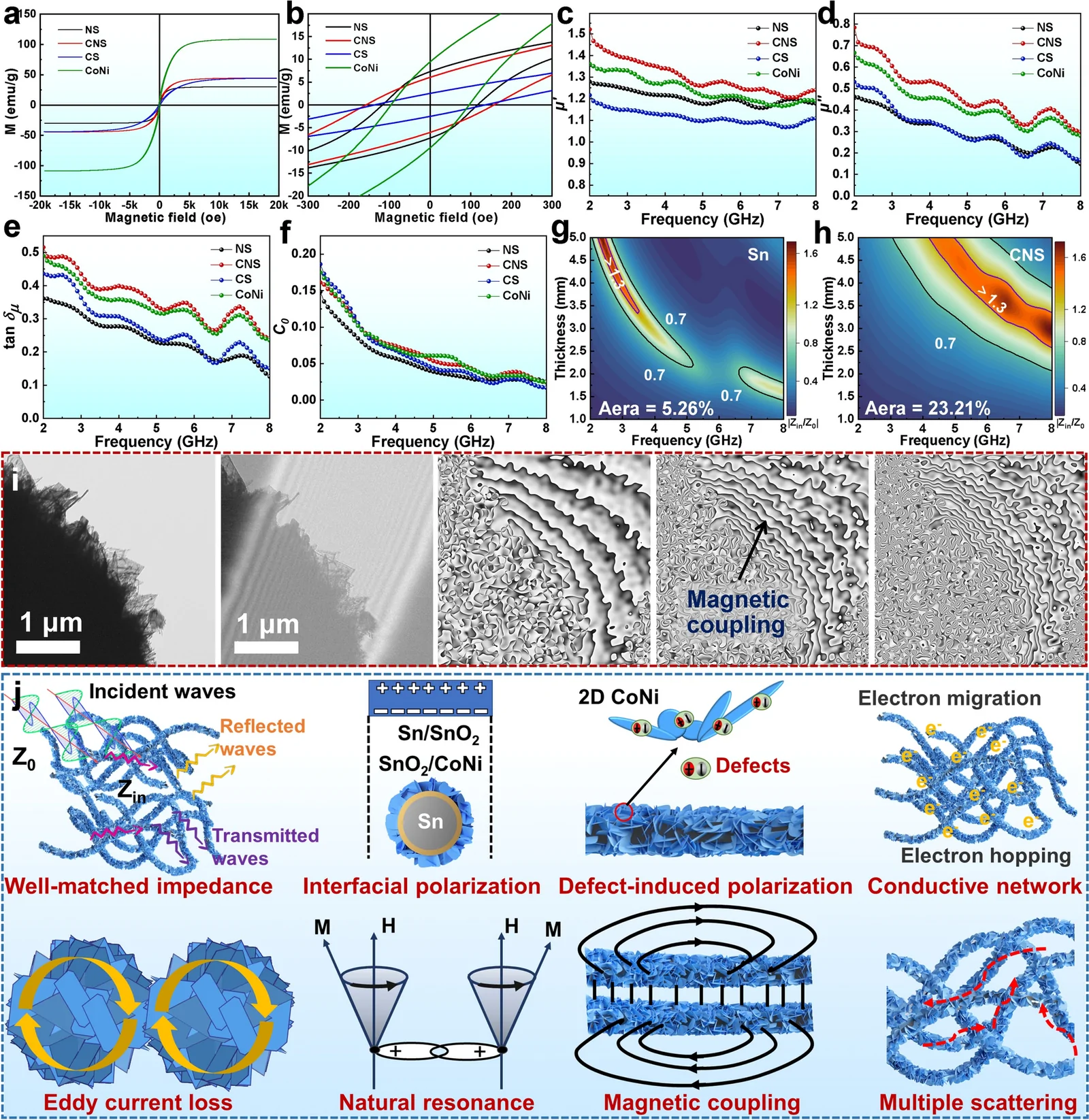
Low-frequency microwave absorption mechanisms: magnetic loss. **a, b** Room-temperature hysteresis loops of samples. **c** *μ*′, **d** *μ*″, **e** tan *δ*_μ_, and **f** Eddy current coefficient (*C*_0_) of CS, NS, CoNi and CNS. 2D representations of |*Z*_in_/*Z*_0_| with different thickness of **g** Sn whisker and **h** CNS. **i** TEM image and corresponding magnetic lines of flux for gradually increasing electromagnetic signals. **j** Schematic of the low-frequency microwave-absorbing mechanisms for the CNS sample

The low-frequency magnetic loss mechanism of CNS was subsequently investigated. According to Snoek formula [54]:

5 $$\left( {\mu_{i} - 1} \right)f_{r} = \frac{1}{3\pi }\gamma M_{s} ,$$

where μ_i_, f_r_, γ, and M_s_ are the initial permeability, natural resonance frequency, gyromagnetic ratio, and saturation magnetization strength, respectively. It can be observed that the product of the μ_i_ and f_r_ is determined by the intrinsic properties of the material, namely the saturation magnetization and the gyromagnetic ratio. The product of μ_i_ and f_r_ is a constant, a relationship known as the Snoek limit. To achieve efficient MA performance in the low-frequency region, it is essential to increase the natural resonance frequency f_r_ of magnetic materials to break through this limitation. According to ferromagnetic resonance theory, the natural resonance can be expressed as [55]:

6 $$2\pi f_{r} = \gamma H_{a} ,$$

7 $$H_{a} = 4\left| {K_{1} } \right|/3\mu_{0} M_{s} ,$$

8 $$K_{1} = \mu_{0} M_{s} H_{c} {/}2,$$

where *H*_a_ is the magnetic anisotropy energy, |K_1_| is the anisotropy coefficient, and *H*_c_ is the coercivity. This reveals a positive correlation between *H*_a_ and *H*_c_; in general, a higher coercivity indicates stronger magnetic anisotropy of the material. Coercivity reflects the intensity of the irreversible magnetization process, and an increase in *H*_c_ suggests the presence of stronger internal magnetic anisotropy. Room-temperature magnetic hysteresis loops were measured for the magnetic samples, as shown in Fig. 5a, b and Table S2. Compared with CS and NS, the CNS sample exhibits a higher *M*_s_ of 44.64 emu/g and the highest *H*_c_ of 160.27 Oe which is 1.6 times that of the CoNi sample (99.73 Oe). In addition, we calculated the |*K*_1_| values of these materials (Table S3) and found that CNS exhibits the highest magnetic anisotropy, whereas NS shows the lowest. This result indicates that shape-induced magnetic anisotropy enhances both M_s_ and H_c_, thereby contributing to higher permeability and resonant loss. Meanwhile, the higher M_s_ and H_c_ result in a larger hysteresis loop area and stronger magnetic loss capability. Correspondingly, the initial values of μ′ and μ″ for the CNS sample are the highest (Fig. 5c, d), confirming that a morphology with strong anisotropy exhibits greater magnetization intensity and higher magnetic permeability, thereby enabling a breakthrough of the Snoek limit.

As the frequency increases, μ′ decreases for all samples, while μ″ fluctuates within a certain range. Notably, the overall decline of μ′ in CNS is more gradual, indicating a higher magnetic energy storage capability compared to other samples. Furthermore, the μ″ curves of all samples exhibit multiple fluctuation peaks across 2–8 GHz, nearly spanning the entire frequency range, indicating the presence of resonance behavior in the low-frequency region. The natural resonance peaks of CNS are more pronounced (with steeper slopes for each peak) and exhibit higher intensities, further confirming the effectiveness of this strategy in enhancing natural resonance losses (Fig. 5d). Meanwhile, the relatively higher μ″ peak intensities indicate stronger magnetic loss capability, consistent with the highest tan δ_μ_ values (Fig. 5e). In addition, magnetic loss in the 2–8 GHz range may also originate from eddy current loss. The eddy current loss coefficient (C_0_) is calculated using the following equation [56]:

9 $$C_{0} = \mu^{\prime \prime } \left( {\mu^{\prime } } \right)^{ - 2} f^{ - 1} .$$

If magnetic loss arises from eddy currents, the value of C_0_ remains constant with varying frequency. It can be seen that all samples exhibit a few small plateau regions, though limited in proportion, indicating that while eddy current loss exists, it is not dominant. Meanwhile, the percentage contributions of magnetic and dielectric loss tangents to the total loss tangent in the 2–8 GHz range were calculated, as shown in Fig. S18. It is indicated that magnetic loss is dominant in the magnetic–dielectric synergistic loss mechanism of the CNS samples in the low-frequency region.

The influence of CoNi modification on the impedance matching characteristics of the whiskers was further investigated. As shown in Fig. S19, the absorption performance of both Sn whiskers and the CNS sample conforms to the quarter-wavelength (λ/4) cancelation model, expressed as [57]:

10 $$t_{m} = \frac{n\lambda }{4} = \frac{nc}{{\left( {4f_{m} \left( {\left| {\mu_{r} \left| \right|\varepsilon_{r} } \right|} \right)^{\frac{1}{2}} } \right)}},\left( {n = 1, 3, 5 \ldots } \right),$$

where *t*_m_ and *f*_m_ are the matching thickness and corresponding frequency, respectively. When *t*_m_ and *f*_m_ satisfy Eq. (10), the phase difference between the incident and reflected waves is 180°, resulting in destructive interference [58]. The results indicate that the simulated t_m_ values agree well with the experimental data. The λ/4 model also explains the shift of the RL peak toward lower frequencies with increasing coating thickness. Moreover, CNS achieves effective absorption of microwaves in the 2.3–8 GHz range at a coating thickness of 1–5 mm, offering a tunable EAB of 5.7 GHz that covers 95% of the low-frequency region—much broader than that of the Sn whiskers. As shown in Fig. S19c, f, the optimal RL_min_ values generally appear at impedance matching values (|*Z*_in_/*Z*_0_|) close to 1, indicating minimal wave reflection [59]. The dielectric constant and magnetic permeability of the samples can be effectively tuned to optimize impedance matching. Figure 5g, h shows that the magnetic modification significantly enlarges the impedance matching region (defined in the low-frequency region as |Z_in_/Z_0_| values between 0.7 and 1.3), increasing the matching area from 5.26% for Sn whiskers to 23.21% for CNS. This demonstrates that the strategy significantly balances the magnetic and dielectric parameters, improving impedance matching and allowing more incident microwaves to penetrate the material for further attenuation.

Traditionally, magnetic metals such as Ni, Co, and CoNi tend to form self-assembled compact aggregates due to their intrinsic magnetism, isotropy, and uniform magnetic domain distribution, as observed under the same synthesis conditions in this study. This issue can be effectively mitigated by modulating the nucleation and growth processes of magnetic materials—e.g., using directional inducers or other heterogeneous substrates. The highly anisotropic 2D layered CoNi nanosheets are well dispersed on the surface of Sn whiskers rather than tightly aggregated, which is conducive to forming a macroscopic 3D magnetic coupling network, thereby enhancing magnetic loss capability. To observe the magnetic field distribution around the CNS sample, off-axis electron holography characterization was conducted, as shown in Fig. 5i. Notably, under low magnetic field intensity, the magnetic flux lines of CNS are relatively sparse. Therefore, an enhanced magnetic field was applied during the holography measurement to more intuitively compare their orientations. The flux lines are highly concentrated and divergent near the tips, indicating denser magnetic domains and higher magnetic energy at the tips. These results show that the anisotropic 2D sheet structure significantly enhances the material’s sensitivity to external electromagnetic fields [54]. Furthermore, strong magnetic coupling is observed between the ends of CNS structures, which contributes to an increased initial permeability and enhanced magnetic loss performance.

Based on the above analyses, the low-frequency MA mechanism of the CNS sample is illustrated in Fig. 5j. First, the in situ grown oxides and the 2D CoNi shell on the surface of Sn whiskers balance the magnetic and dielectric parameters, greatly optimizing impedance matching in the low-frequency region. Second, the high aspect ratio and large specific surface area of the 1D Sn whiskers provide abundant interfacial coupling sites, creating a larger interfacial area. The SnO_2_ interlayer and CoNi shell introduced through interface engineering possess different work functions, leading to asymmetric charge distribution across the heterointerface. This creates an internal electric field that intensifies interfacial polarization loss, dissipating microwaves. Additionally, the CoNi nanosheets contain abundant point defects, dislocations, and lattice distortions, which serve as charge-trapping centers and induce local electric field distortions. When subjected to an external electric field, the separation of positive and negative charge centers is facilitated, giving rise to defect-induced polarization that dissipates electromagnetic energy. Meanwhile, electrons are driven by the external field to flow radially along the 1D conductive Sn core of CNS and quickly spread through the interconnected 3D network. Hopping conduction among these conductive networks further enhances conductive loss. The incident microwaves are also repeatedly reflected and scattered within the network and between CoNi sheets, leading to effective energy dissipation. Notably, the CoNi shell imparts magnetic loss characteristics to the Sn whiskers, including eddy current loss, natural resonance, and magnetic coupling effects. Most importantly, the nanosheet-like structure enhances magnetic anisotropy and elevates natural resonance levels, thereby breaking the Snoek limit. The resulting macroscopic magnetic coupling effect further strengthens low-frequency microwave attenuation and significantly broadens the EAB.

### Low-Frequency MA and Thermally Conductive CNS/TPU Film

**Fig. 6:**
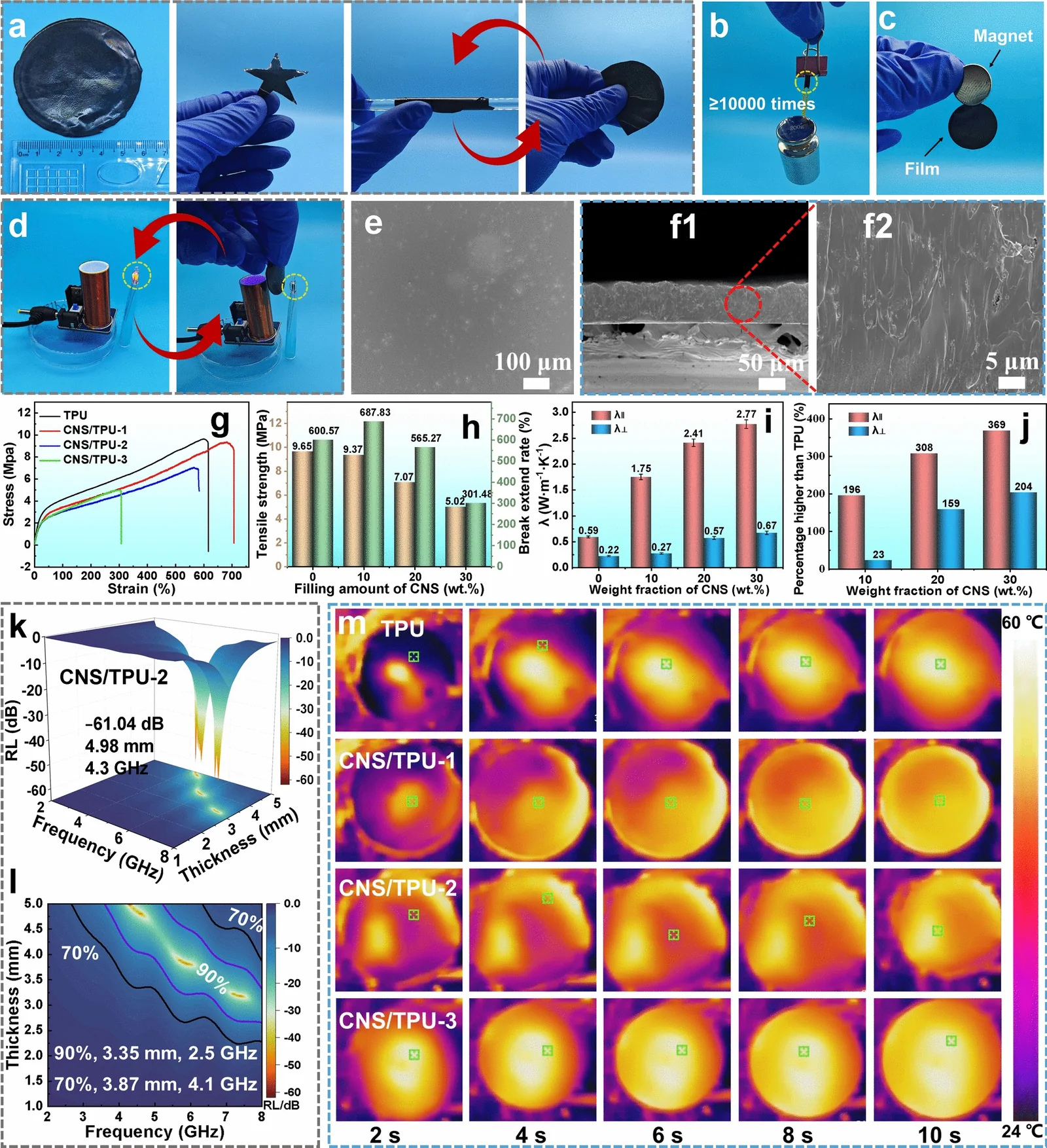
Multifunctional CNS/TPU films. **a** Optical photographs of customizable shaped CNS/TPU-2 film and its curling and recovery. Demonstration of **b** tensile and **c** magnetic properties and **d** the electromagnetic protection (i.e., shielding of electromagnetic signals from the Tesla coil to shut off a light bulb) of the CNS/TPU-2 film. SEM images of the **e** surface and **f** cross section of the CNS/TPU-2 film. **g** Stress–strain curves and **h** Tensile strength and elongation at break of CNS/TPU films. **i** In-plane and through-plane thermal conductivity of films with different filling amount of CNS. **j** Percentage improvement in in-plane and through-plane thermal conductivity of CNS/TPU films over pure TPU film. **k**–**l** Low-frequency MA properties. **m** Infrared thermal images of TPU films with different CNS fillings for heating up on a hot plate at 60 °C

Given the promising low-frequency MA performance of the absorber, additional functional attributes such as mechanical robustness and thermal management should also be considered for practical applications. Therefore, a series of TPU-based composite absorber films with varying contents of CNS were fabricated (denoted as CNS/TPU) via ultrasonic/vibrational dispersion followed by vacuum drying. Digital photographs show that the diameter of the resulting CNS/TPU films can be well controlled, reaching sizes over 5 cm. Moreover, the shape of the films can be customized through mold design or post-fabrication cutting to adapt to various application scenarios, such as conformal coating on the surface of electronic components. These films also exhibit excellent flexibility and can be bent or rolled into arbitrary shapes and recover their original form (Video S1). Figure S20 highlights the lightweight nature of the TPU-based films. The density of the pure TPU film is 1.1 g cm^−3^, and incorporating a moderate amount of CNS does not significantly increase the density. For example, a film with 20 wt% CNS (CNS/TPU-2) exhibits a density of only 1.2 g cm^−3^. As shown in Fig. 6b, the CNS/TPU-2 film exhibits remarkable tensile strength, capable of supporting a load 10,000 times its own weight without breaking (Video S2). Due to the excellent soft magnetic properties of CNS, the CNS/TPU-2 film is easily attracted by a magnet (Fig. 6c and Video S3). To validate the electromagnetic protection performance of the magnetic composite film, a Tesla coil experiment was conducted using a small bulb [60]. As shown in Fig. S21, the presence or removal of a pure TPU film has little effect on the bulb. In contrast, Fig. 6d shows that the CNS/TPU-2 film can effectively block electromagnetic signals, causing the bulb to turn off; removing the film allows the bulb to light up again, further demonstrating the excellent electromagnetic shielding capability of the CNS filler (Video S4). Figures 6e and S22 display the surface morphologies of films with varying CNS contents. Once the CNS loading exceeds 40 wt%, open porosity emerges, which could be detrimental to practical applications. The cross-sectional SEM images in Figs. 6f and S23 reveal that the film thickness ranges from 50 to 150 μm, and films with ≤ 30 wt% CNS exhibit a dense structure. Based on this, tensile tests were conducted on films containing 0 wt%–30 wt% CNS. The results indicate that adding 10 wt% CNS does not compromise the tensile strength of TPU and even slightly improves the elongation at break, possibly due to mechanical interlocking and crack deflection. When the CNS content exceeds 20 wt%, both the tensile strength and elongation at break begin to decline due to the increased porosity. Notably, CNS/TPU-2 still retains a tensile strength above 7 MPa and an impressive elongation of 550%, while CNS/TPU-3 shows a significant deterioration in mechanical performance, limiting its application in flexible electronics. Further EDS mapping analysis of CNS/TPU-2 (Fig. S24) confirms that CNS is uniformly distributed throughout the TPU matrix.

During operation, electronic devices generate a large amount of waste heat, which can accumulate and impair performance or even cause damage. Therefore, excellent thermal conductivity is critical for MA materials used in low-frequency applications. To this end, we investigated the thermal conductivity of films with different CNS contents (Fig. 6i, j). The results show that both the in-plane (λ_∥_) and through-plane (λ_⊥_) thermal conductivities increase with higher CNS loading, with λ_∥_ showing a more pronounced increase. The CNS/TPU-2 film achieves λ_∥_ and λ_⊥_ values of 2.41 and 0.57 W m^−1^ K^−1^, respectively—approximately 3.1 and 1.6 times higher than those of pure TPU (0.59 and 0.22 W m^−1^ K^−1^, respectively). When the CNS content reaches 30 wt%, CNS/TPU-3 shows maximum λ_∥_ and λ_⊥_ values of 2.77 and 0.67 W m^−1^ K^−1^. These enhancements are attributed to the conductive 1D Sn core and the CoNi alloy shell of CNS, as well as the increased overlap between whiskers at higher loadings, which facilitates the formation of continuous thermal pathways. This transition from 1 to 3D heat conduction networks greatly improves the thermal conductivity of the CNS/TPU composites. Electromagnetic parameters and low-frequency MA performance of CNS/TPU films were also evaluated. The results (Figs. S25 and S26) show that MA performance initially improves and then declines with increasing CNS content. Among them, CNS/TPU-2 exhibits the best performance, with an RL_min_ value of − 61.04 dB in the low-frequency region, an EAB-90 of 2.5 GHz, and an EAB-70 of 4.1 GHz, as shown in Fig. 6k, l. Figure S27 also indicates that the film maintains excellent MA performance over the 2–18 GHz range, with EAB-90% exceeding 4 GHz at a thickness of only 1.75 mm, further validating the effectiveness of the magnetic–dielectric synergistic strategy. In addition, Fig. S28 presents the electrical conductivity and conductive loss characteristics of composite films with different CNS loadings. The results indicate that the conductivity exhibits a pronounced nonlinear increase with increasing whisker content, accompanied by a significant rise in the proportion of conductive loss (ε_c_″/ε″). In the low-frequency region, conductive loss accounts for the major contribution to dielectric loss, whereas polarization loss becomes dominant as the frequency increases.

To further assess the suitability of the film for practical low-frequency applications, we explored the influence of CNS content on heat conduction and dissipation. The films were placed on a 60 °C hot stage and monitored using an infrared camera (Fig. 6m). The CNS/TPU films reached thermal equilibrium with the hot stage more rapidly than the pure TPU film, and the rate increased with higher CNS loading, consistent with the thermal conductivity results (Fig. S30a). Subsequently, the films were removed from the hot stage, and their cooling process was recorded (Fig. S29). Both CNS/TPU-2 and CNS/TPU-3 cooled to 25 °C within 12 s (Fig. S30b), demonstrating excellent heat dissipation performance, which is attributed to the increased thermal diffusivity, while the density and specific heat capacity show only minor variations. Collectively, the results demonstrate that the CNS/TPU composite films possess a unique combination of flexibility, stretchability, thermal conductivity, and low-frequency MA performance, rendering them promising candidates for electromagnetic protection of communication electronic devices.

## Conclusions

Sn whiskers derived from the mechanochemical decomposition of Ti_2_SnC MAX phase were employed as 1D templates with excellent electrical and thermal conductivity to construct magnetic–dielectric composite materials for low-frequency MA. The in situ grown SnO_2_ layer on the Sn whiskers formed a metal/semiconductor hybrid structure, which further enhanced low-frequency dielectric loss. On this basis, CoNi magnetic alloy nanosheets were grown on the surface of the 1D Sn/SnO_2_ via a hydrothermal method, forming a 2D/1D hierarchical CoNi@SnO_2_@Sn (CNS) heterostructure. The CNS-2 sample achieved a broad EAB-70 of 4.0 GHz (at 2.61 mm) in the low-frequency region, covering the entire C-band. It also exhibited an RL_min_ value of -62.29 dB at a thickness of only 2.44 mm, outperforming most existing low-frequency absorbers. The enhanced magnetic anisotropy of CoNi nanosheets promotes natural resonance, and the induced magnetic coupling synergistically improves magnetic loss. Meanwhile, the Sn core and its interconnected conductive network contributed to conduction loss. The abundant Sn/SnO_2_ and CoNi/SnO_2_ metal/semiconductor heterointerfaces, together with defects in the alloy nanosheets, promoted strong dielectric polarization. Furthermore, the CNS filler was incorporated into a TPU matrix to fabricate flexible CNS/TPU composite films. The CNS/TPU-2 film containing 20 wt% CNS exhibited a remarkable RL_min_ value of -61.04 dB and an EAB-70 of 4.1 GHz in the low-frequency range. Its in-plane and through-plane thermal conductivities reached 2.41 and 0.51 W m^−1^ K^−1^, respectively, which are 4.1 and 2.6 times those of the pure TPU film. This magnetic–dielectric synergistic strategy provides new insights into enhancing low-frequency MA performance. The flexible CNS/TPU films exhibit both excellent low-frequency MA and high thermal conductivity, making them promising candidates for applications in 5G communications and flexible electronics.

## Supplementary Information

Below is the link to the electronic supplementary material.

Supplementary file1 (MP4 2194 KB)

Supplementary file2 (MP4 1022 KB)

Supplementary file3 (MP4 1531 KB)

Supplementary file4 (MP4 828 KB)

Supplementary file5 (DOCX 15495 KB)
