# Supplementary material for: Magnetic–Dielectric Synergy in One-Dimensional Metal Heterostructures for Enhanced Low-Frequency Microwave Absorption
Source: Nanomicro Lett. 2026 Jan 5;18:155. doi: 10.1007/s40820-025-01995-8 (PMC12765767; doi:10.1007/s40820-025-01995-8)
Supplement: Supplementary file 5 — Supplementary file5 (DOCX 15495 KB) [file 40820_2025_1995_MOESM5_ESM.docx]

Supporting Information for

**Magnetic-Dielectric Synergy in One-Dimensional Metal Heterostructures for Enhanced Low-Frequency Microwave Absorption**

Feiyue Hu^1#^, Peigen Zhang^1#^*, Pei Ding^1^, Shuo Zhang^1^, Bingbing Fan^2, 3^, Ali Saffar Shamshirgar^4^, Wei Zheng^1^, Wenwen Sun^1^, Longzhu Cai^5^, Haijiao Xie^6^, Qiyue Shao^1^, Johanna Rosen^4^*, and ZhengMing Sun^1^*

^1^ State Key Laboratory of Engineering Materials for Major Infrastructure, School of Materials Science and Engineering, Southeast University, Nanjing 211189, P. R. China

^2^ School of Materials Science and Engineering, Zhengzhou University, Zhengzhou 450001, P. R. China

^3^ Department of Engineering, Faculty of Environment, Science and Economy, University of Exeter, Exeter EX4 4QF, United Kingdom

^4^ Materials Design Division, Department of Physics, Chemistry and Biology (IFM), Linköping University, SE-581 83, Linköping, Sweden

^5^ State Key Laboratory of Millimeter Waves, School of Information Science and Engineering, Southeast University, Nanjing 210096, P. R. China

^6^ Hangzhou Yanqu Information Technology Co., Ltd, Hangzhou 310003, P. R. China

^#^ Feiyue Hu and Peigen Zhang contribute equally to this paper.

* Corresponding authors. E-mail: [zhpeigen@seu.edu.cn](mailto:zhpeigen@seu.edu.cn) (Peigen Zhang), [johanna.rosen@liu.se](mailto:johanna.rosen@liu.se) (Johanna Rosen) and [zmsun@seu.edu.cn](mailto:zmsun@seu.edu.cn) (ZhengMing Sun)

# S1 RCS simulation

CST Studio Suite 2020 was employed to simulate the radar cross section (RCS) of the microwave absorber, considering the realistic far-field response of the MA materials. A widely adopted metal-back configuration was used, consisting of a dual-layer square specimen (20 × 20 cm^2^). The top layer, 2.44 mm thick, served as the absorbing layer, while the bottom layer was a 1.0 mm perfect electric conductor (PEC). The sample/PEC bilayer was positioned on the X–O–Y plane, with linearly polarized plane waves incident along the +Z to -Z direction and the electric field polarized along the X-axis. Open boundary conditions were applied in all directions, and the field monitor frequency was set to 6.6 GHz. In addition, to further confirm the broadband absorption characteristics of the CNS samples, low-frequency RCS signals at 2.5, 3.5, 5.0, and 8.0 GHz were also simulated. The scattering directions were defined conventionally in spherical coordinates using θ (theta) and φ (phi). The RCS values can be described as follows:

δ (dB m^2^) = 10 lg((4π*S*/λ^2^)|E_s_/E_i_|)^2^ (S1)

Herein, S, λ, E_s_ and E_i_ symbolize the area of the target object simulation model, the wavelength of EMW, the electric field intensity of the scattered wave and the incident wave, respectively.

# S2 Calculation details

Slab model of Co(111) 2×6×1 supercell with four stoichiometric layers was built based on Face Center Cubic (FCC) Co bulk structure. Slab model of Ni(111) 2×6×1 supercell with four stoichiometric layers was built based on FCC Ni bulk structure. Slab model of CoNi(111) with four stoichiometric layers was built based on Co(111) 2×6×1 supercell. Slab model of Sn(100) 2×4×1 supercell with two stoichiometric layers was built based on Sn bulk structure. Slab model of SnO_2_(110) 4×2×1 supercell with three stoichiometric layers was built based on rutile SnO_2_ bulk structure. Slab model of SnO_2_(110)/Co(111) was built based on SnO_2_(110) 1×1×2 supercell and Co(111) 2×6×1 supercell. Slab model of SnO_2_(110)/Ni(111) was built based on SnO_2_(110) 1×1×2 supercell and Ni(111) 2×6×1 supercell. Slab model of SnO_2_(110)/CoNi(111) was built based on SnO_2_(110) 1×1×2 supercell and CoNi(111). Slab model of Sn(100)/SnO_2_(110) was built based on Sn(100) 2×4×1 supercell and SnO_2_(110) 4×2×1 supercell. In slab model, thickness along c direction was set at 40Å to avoid weak interactions between images.

In DFT calculations, structural optimizations were performed by Vienna *Ab-initio* Simulation Package (VASP) with the projector augmented wave (PAW) method. The exchange-functional was treated using the Perdew-Burke-Ernzerhof (PBE) functional, in combination with the DFT-D3 correction. Cut-off energy of the plane-wave basis was set as 450 eV. For optimization of lattice size of Co(111), Ni(111), CoNi(111), Co(111)/SnO_2_(110), Ni(111)/SnO_2_(110) and CoNi(111)/SnO_2_(110), the Brillouin Zone integration was performed with a Monkhorst *k*-point sampling of 2×1×1. For optimization of lattice size of Sn(100), SnO_2_(110) and SnO_2_(110)/Sn(100), the Brillouin Zone integration was performed with a *k*-point sampling of 2×2×1. The self-consistent calculations applied a convergence energy threshold of 10^-5^ eV. The equilibrium geometries were optimized with maximum stress on each atom within 0.02 eV Å^‑1^. Spin polarization method was used to describe magnetism of slab models. Isosurface level of charge density difference was set at 0.02 e Å^-3^. Work functions of slab models were calculated by GGA-PBE functional, and then treated by vaspkit interface.

# S3 Supplementary Figures and Tables


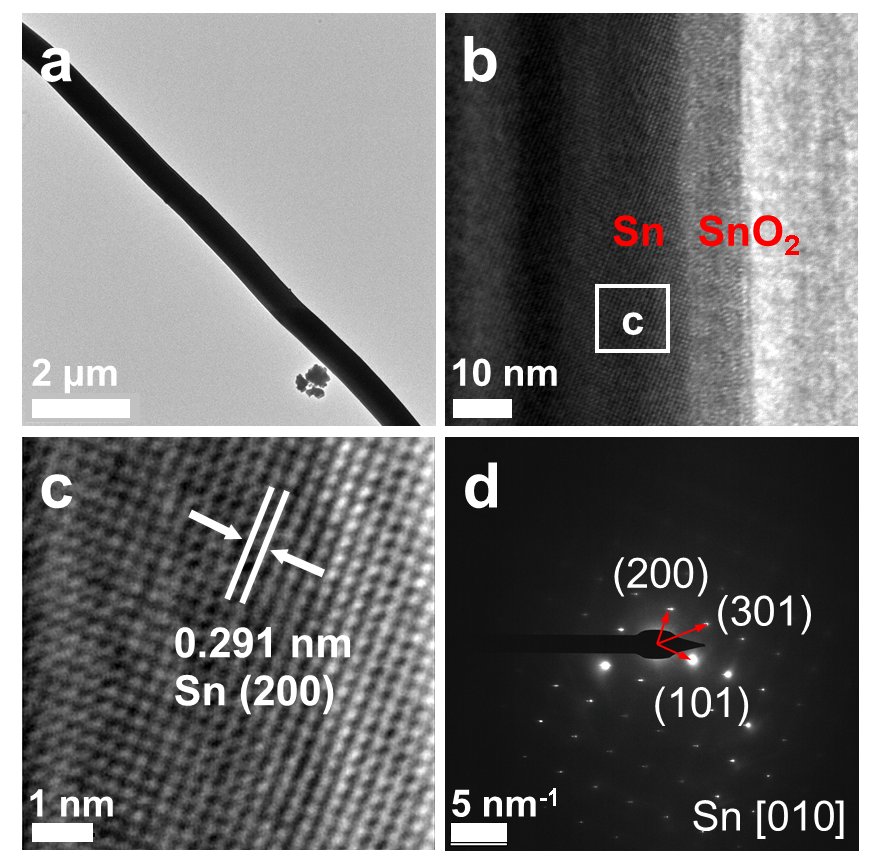


**Fig. S1 a** TEM image, **b, c** HRTEM image and **d** SAED pattern of Sn whisker


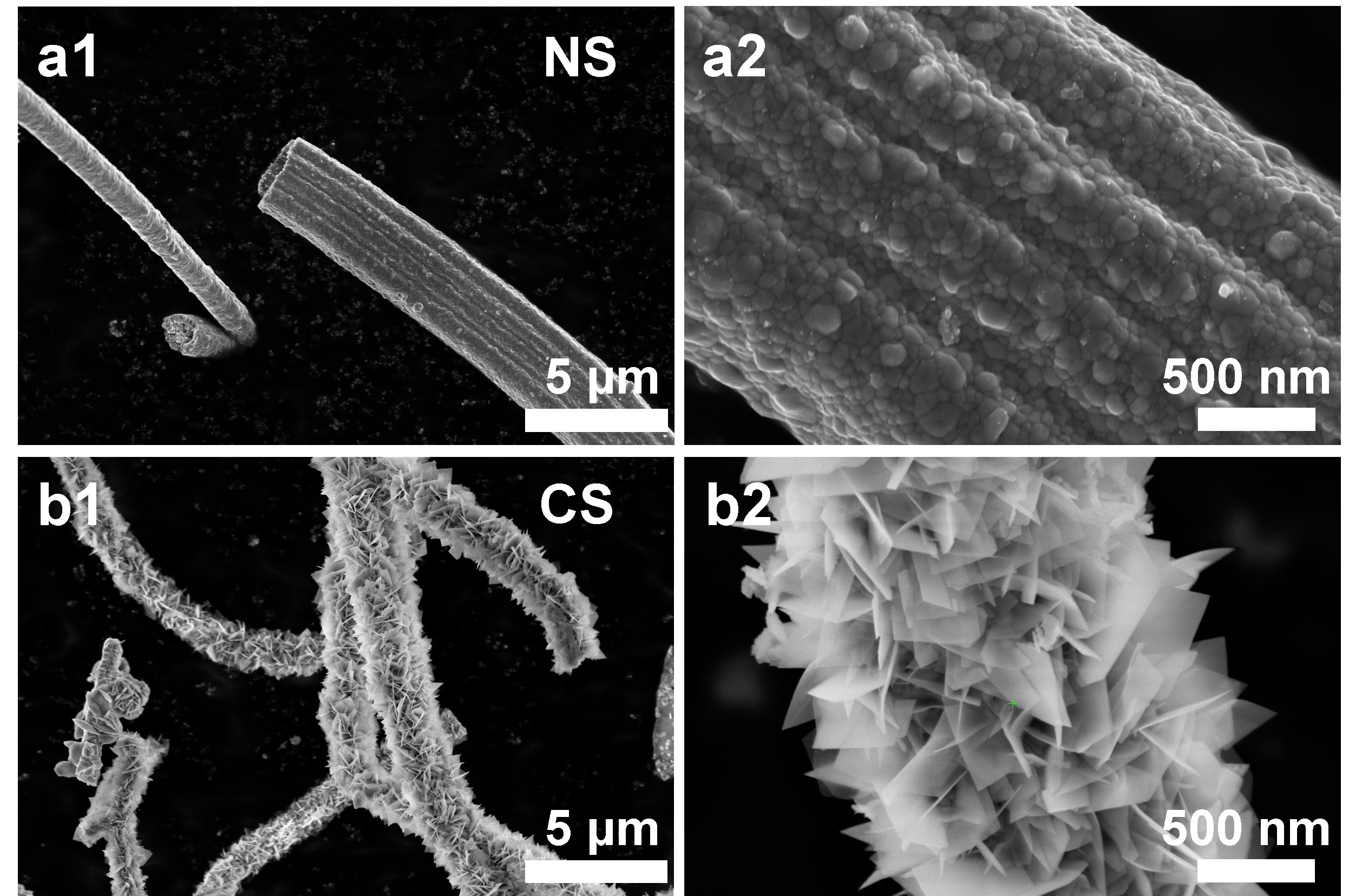


**Fig. S2** SEM images of **a** NS and **b** CS


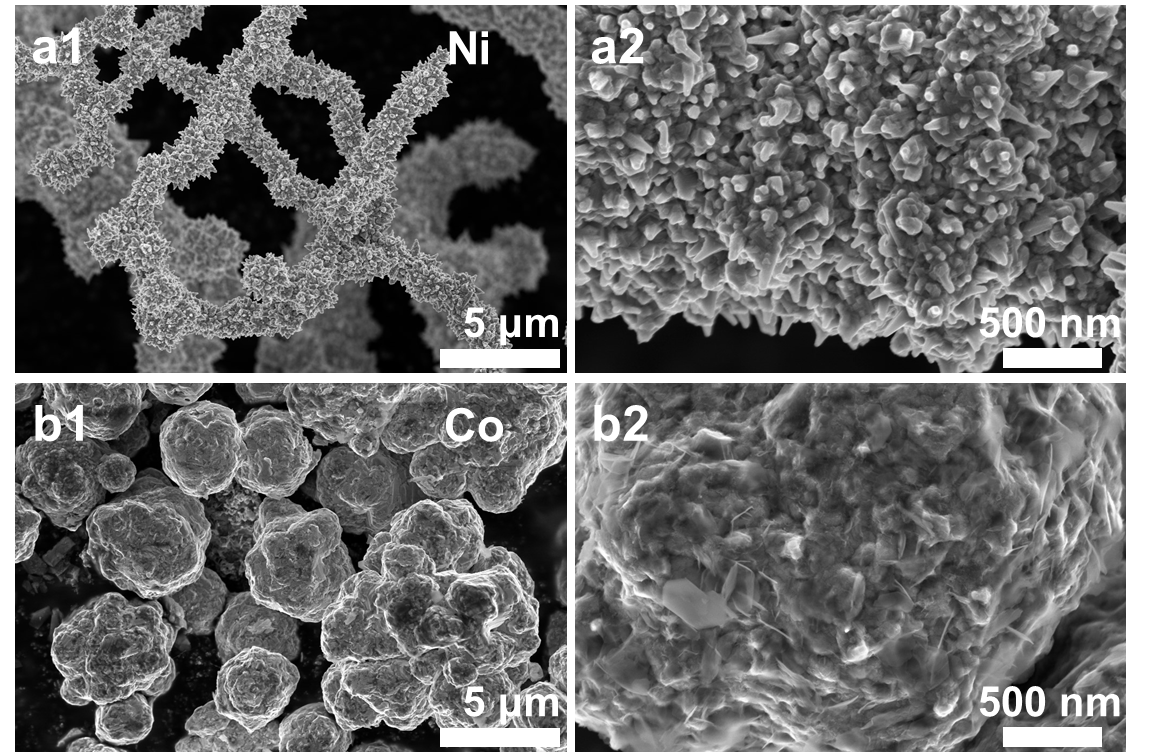


**Fig. S3** SEM images of **a** Ni and **b** Co


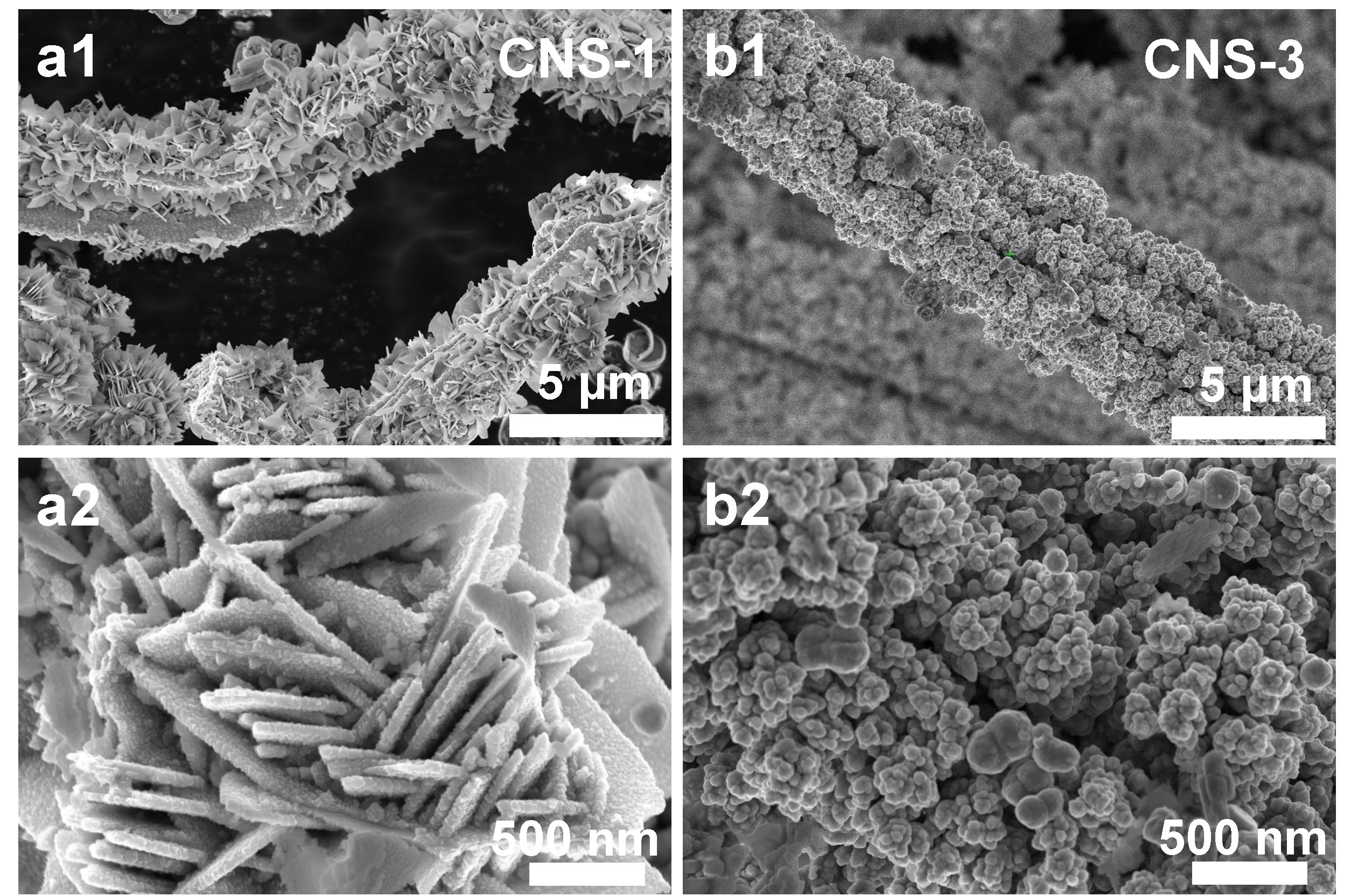


**Fig. S4** SEM images of **a** CNS-1, and **b** CNS-3


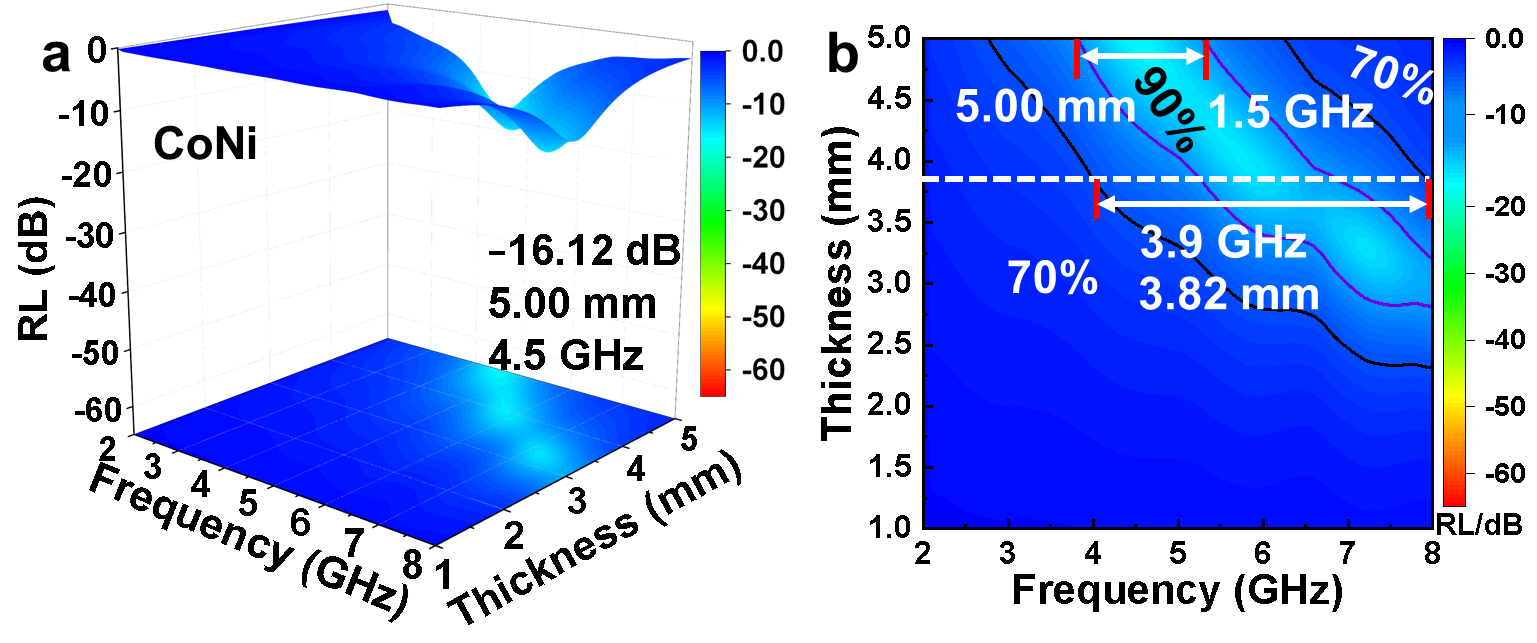


**Fig. S5 a** The RL value and **b** EAB of Sn whisker at low-frequency


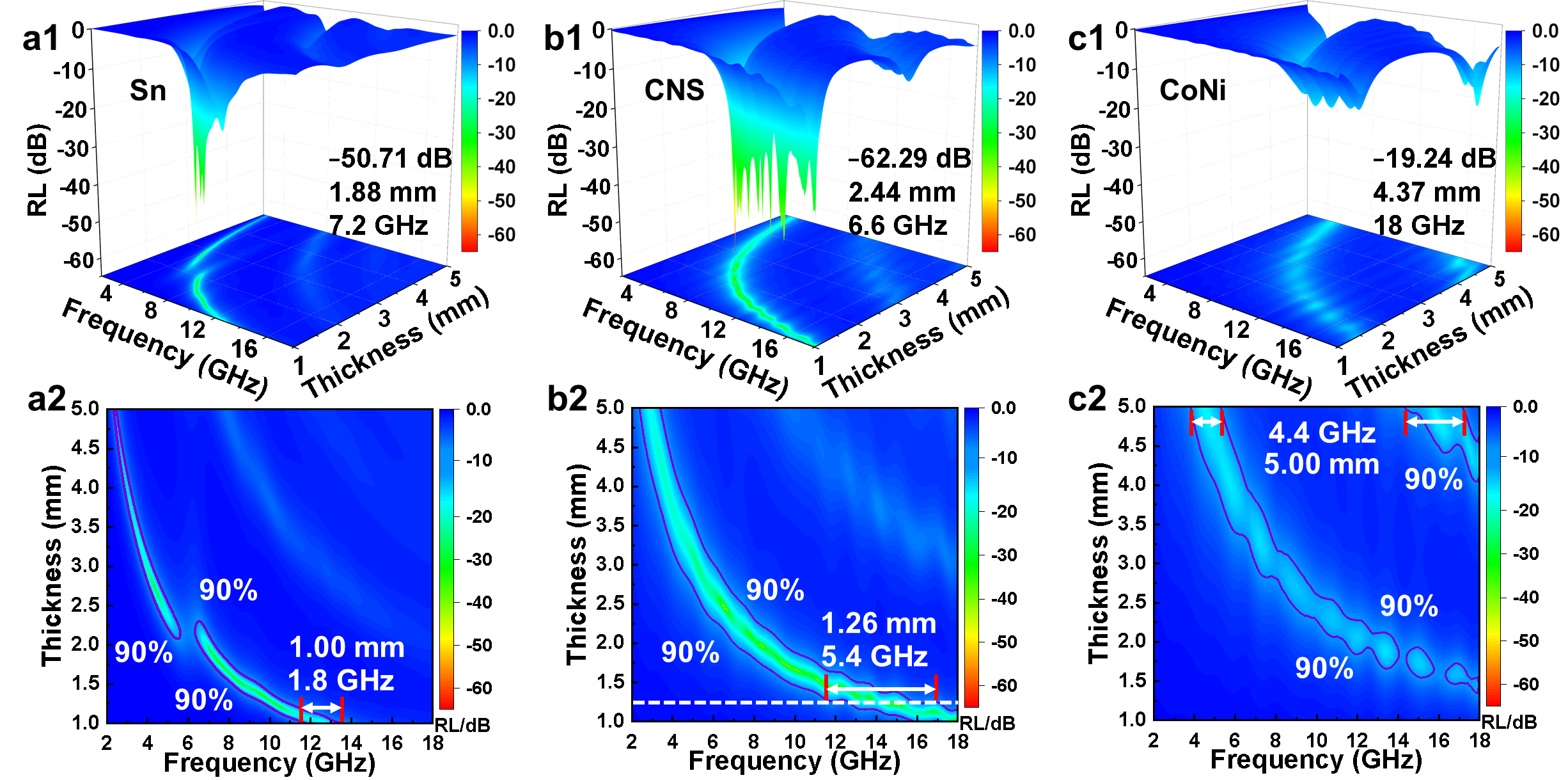


**Fig. S6** The RL value and EAB of **a** Sn whisker, **b** CNS and **c** CoNi at 2-18 GHz


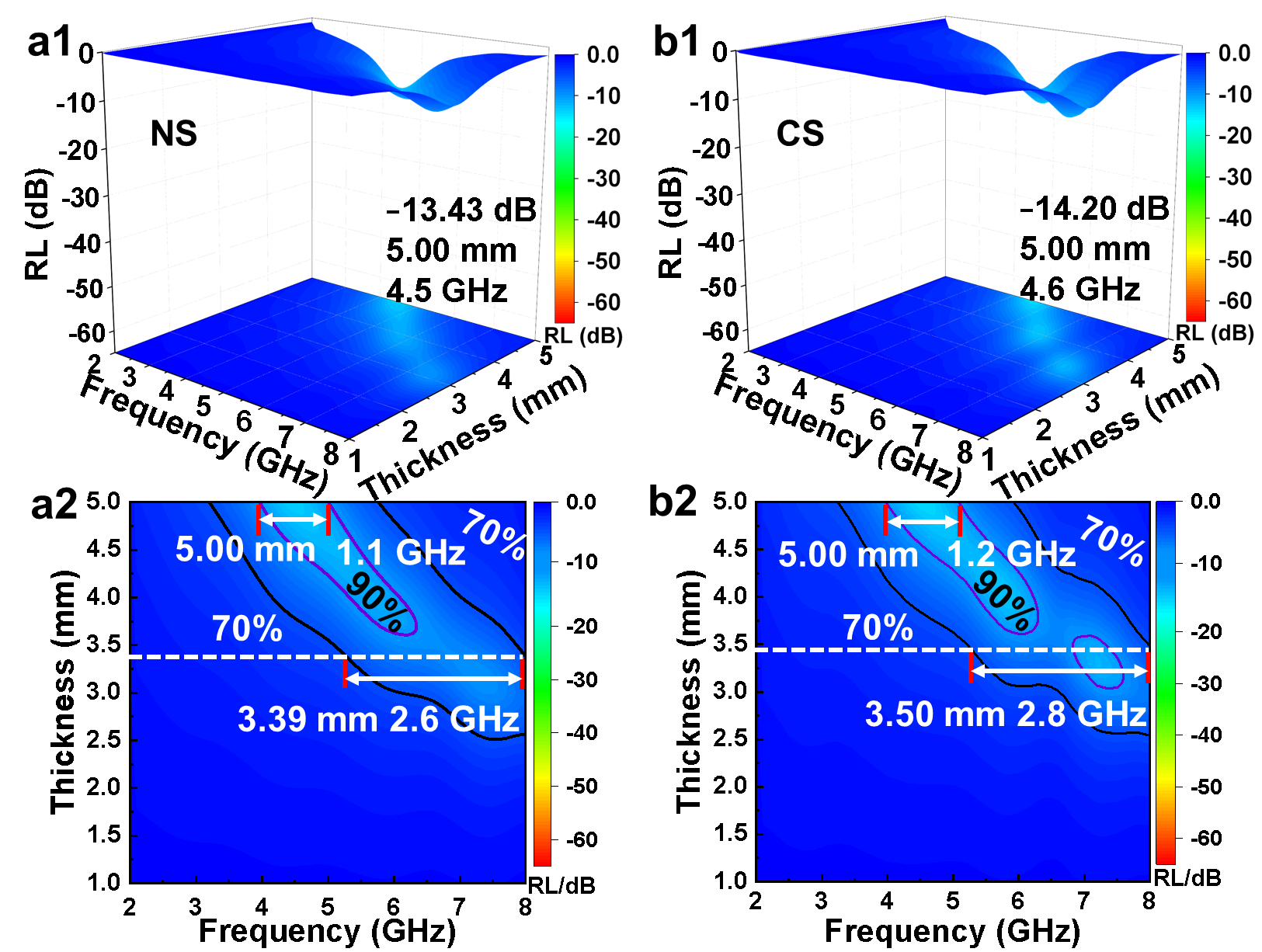


**Fig. S7** The RL value and EAB of **a** NS and **b** CS at low-frequency


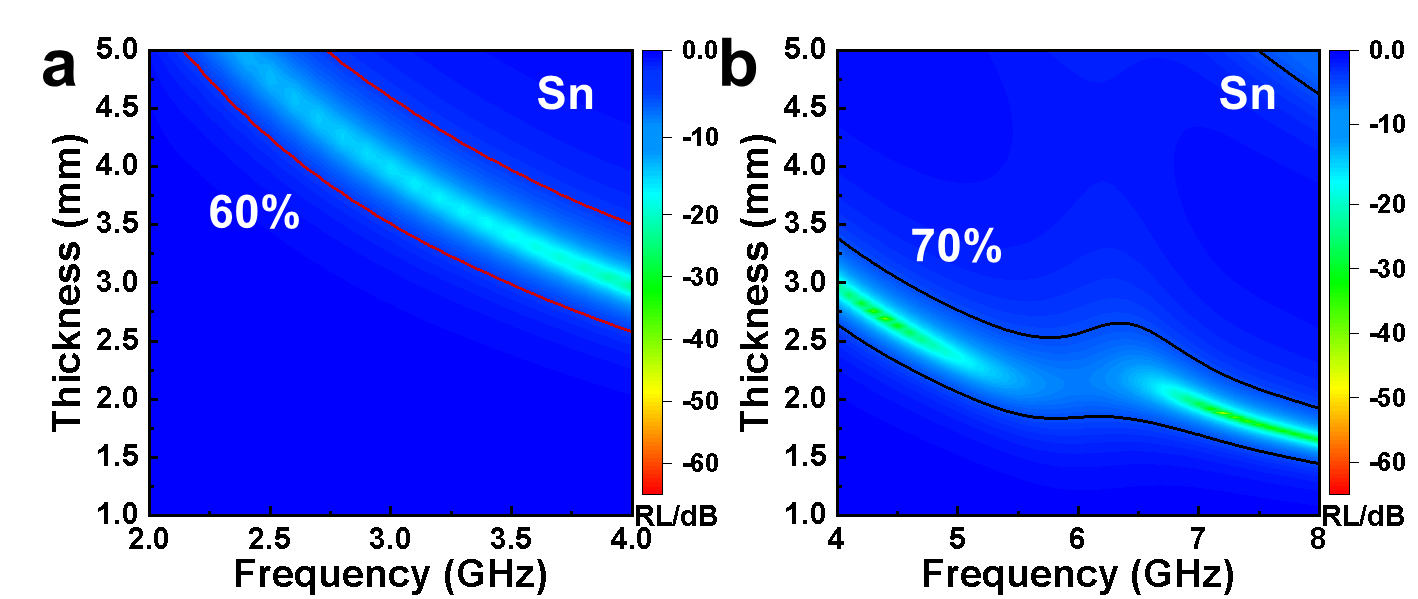


**Fig. S8** The EAB of Sn whisker at **a** S band (EAB-60) and **b** C band (EAB-70)


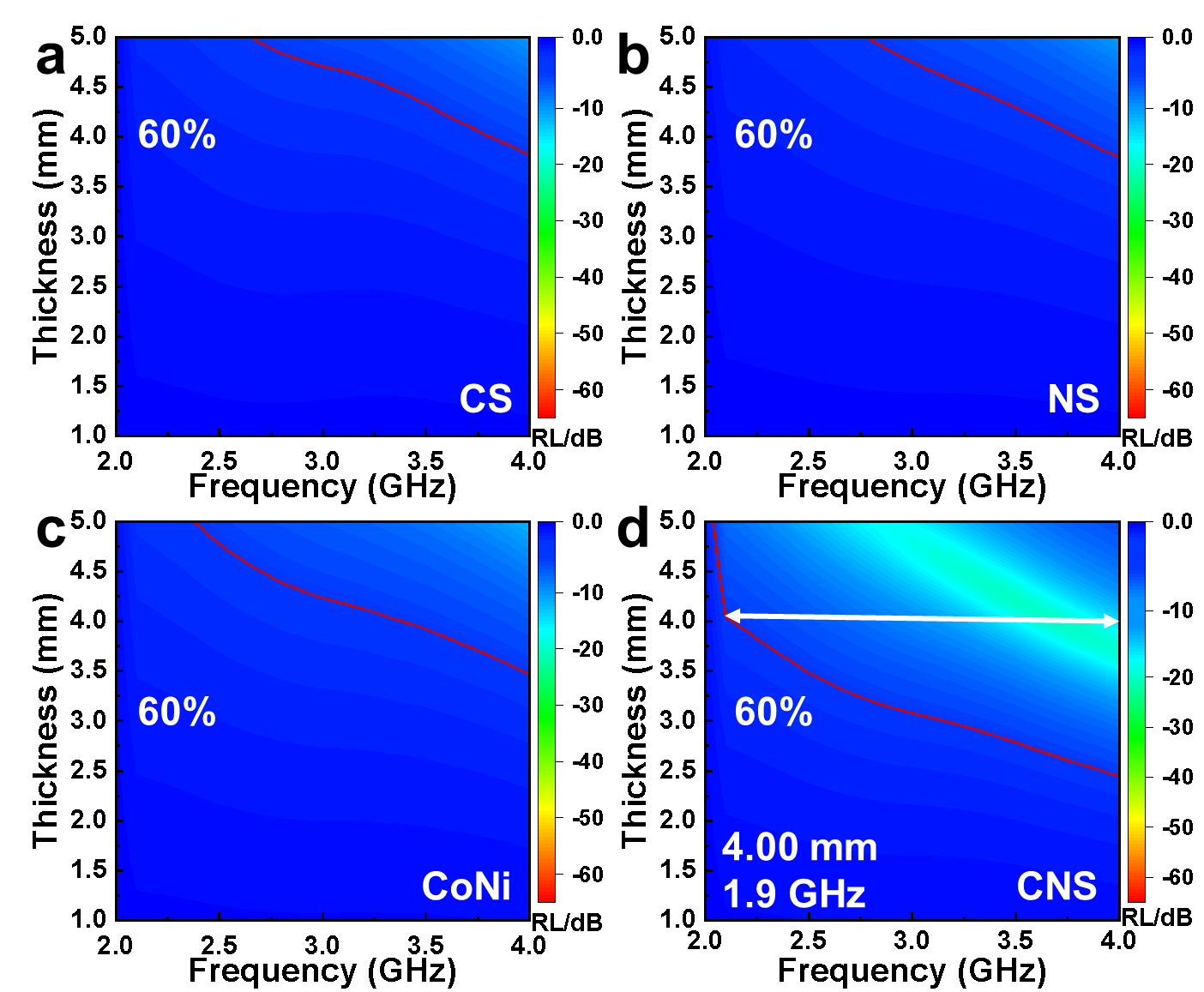


**Fig. S9** The EAB-60 of **a** CS, **b** NS, **c** CoNi, and **d** CNS at S band


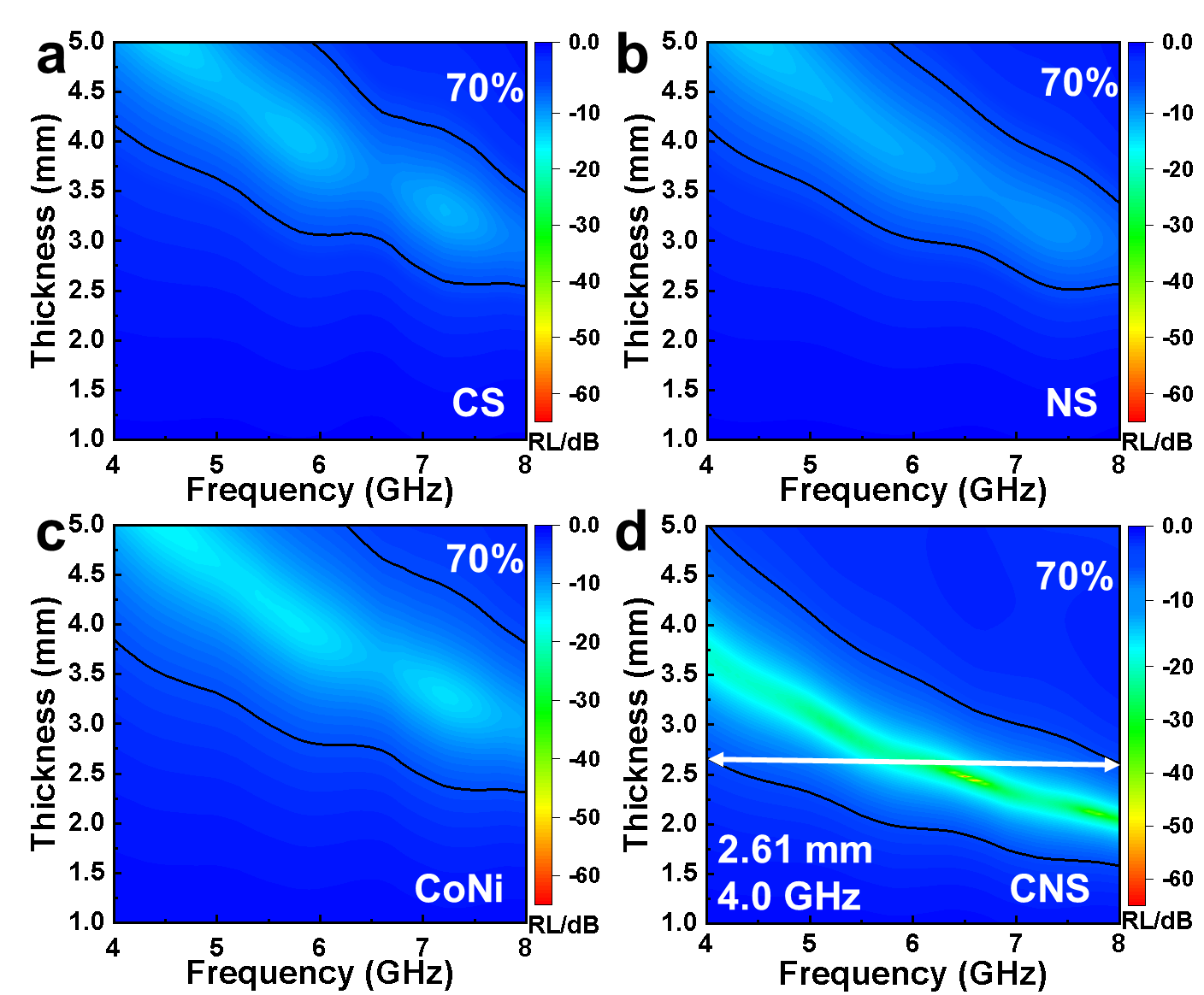


**Fig. S10** The EAB-70 of **a** CS, **b** NS, **c** CoNi, and **d** CNS at C band


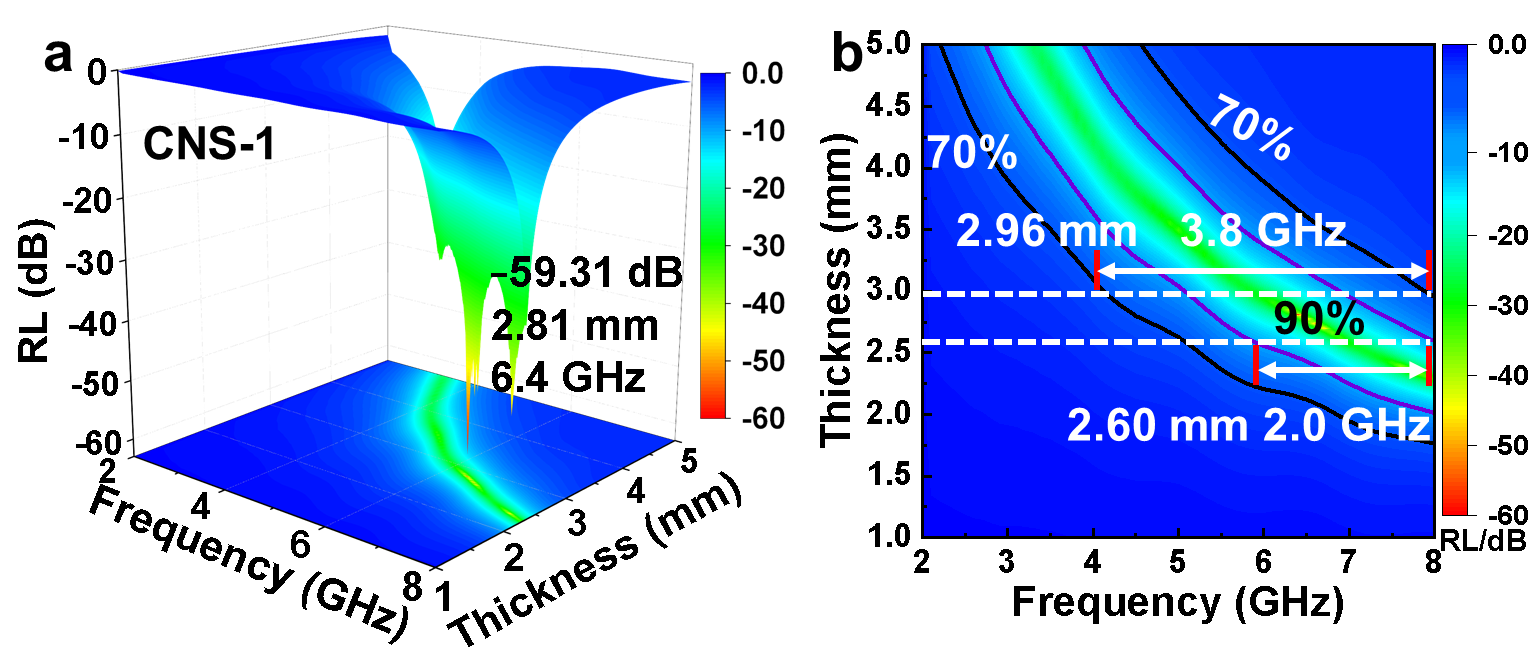


**Fig. S11. a** The RL value and **b** EAB of CNS-1 at low-frequency.


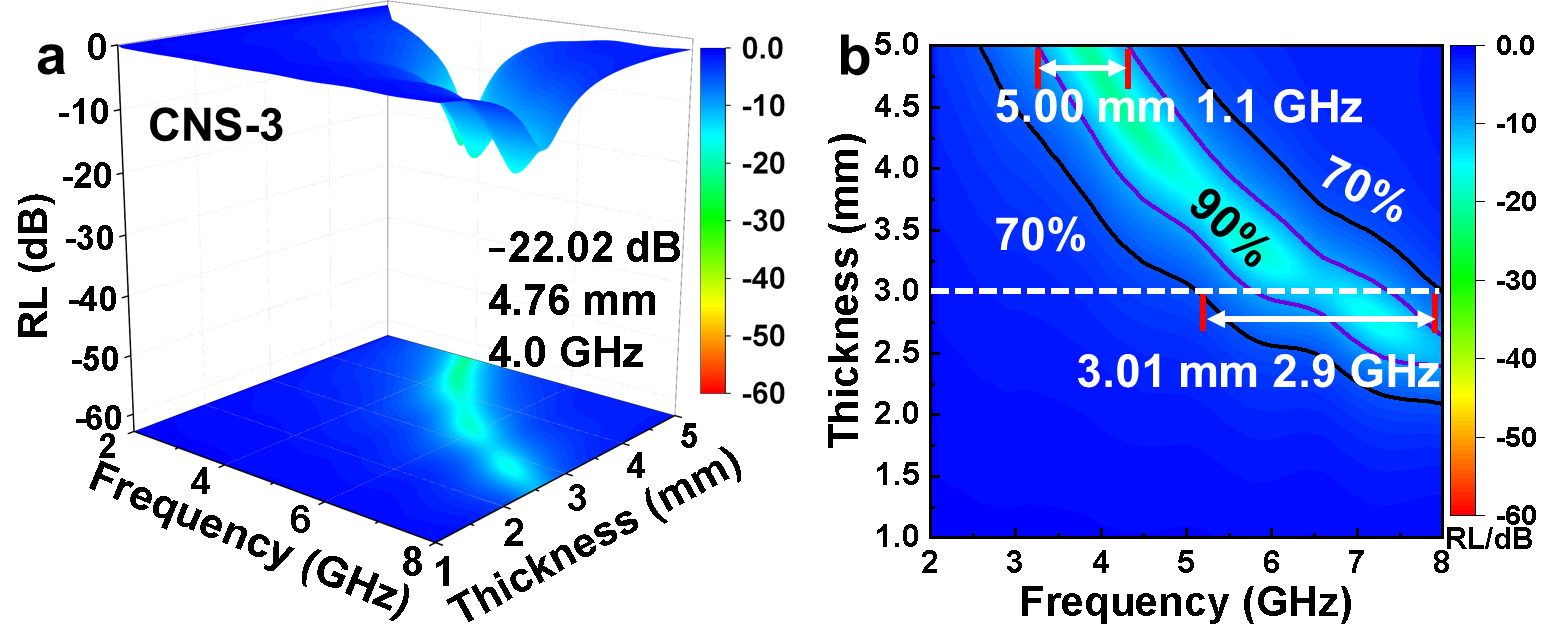


**Fig. S12 a** The RL value and **b** EAB of CNS-3 at low-frequency


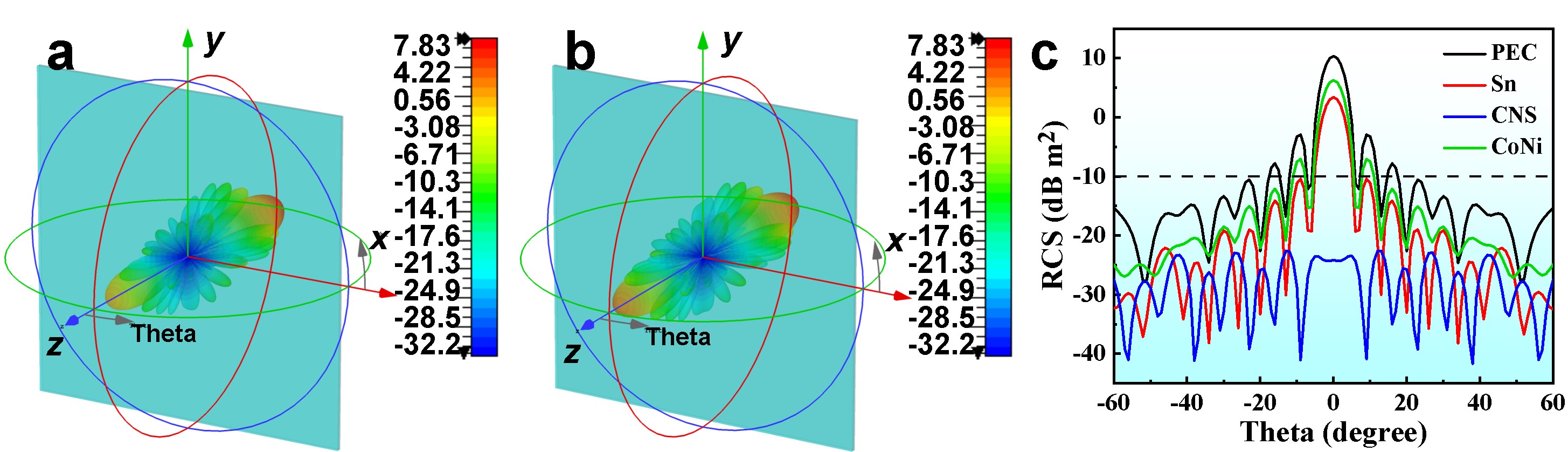


**Fig. S13** PEC plate covered with **a** Sn and **b** CoNi at 6.6 GHz. **c** 2D RCS plots of PEC Plate and sample-coated PEC plates at 6.6 GHz


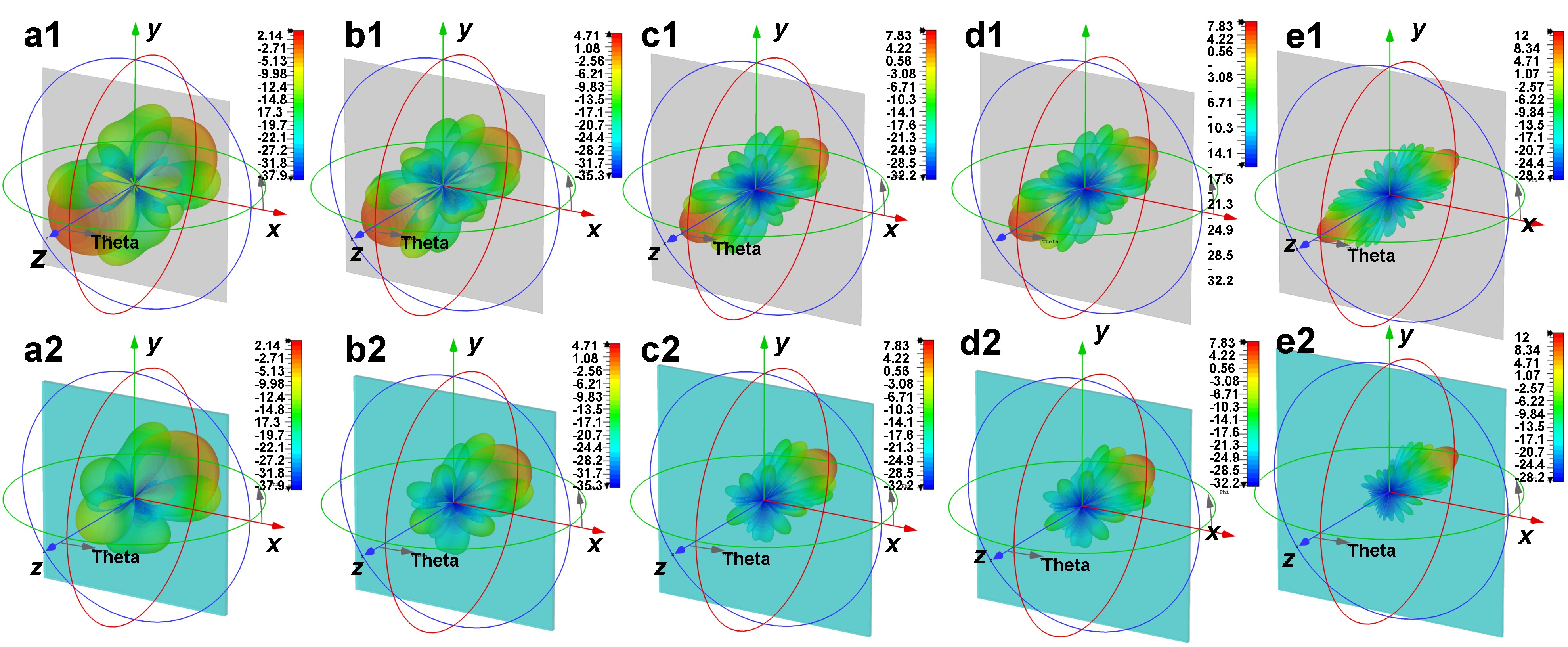


**Fig. S14** 3D RCS plots of PEC and CNS-coated PEC plates at different low frequency points for **a** 2.5 GHz, **b** 3.5 GHz, **c** 5 GHz, **d** 6.6 GHz, and **e** 8 GHz, respectively

**Fig. S15** Work functions for Co, Ni, CoNi, SnO_2_, and Sn


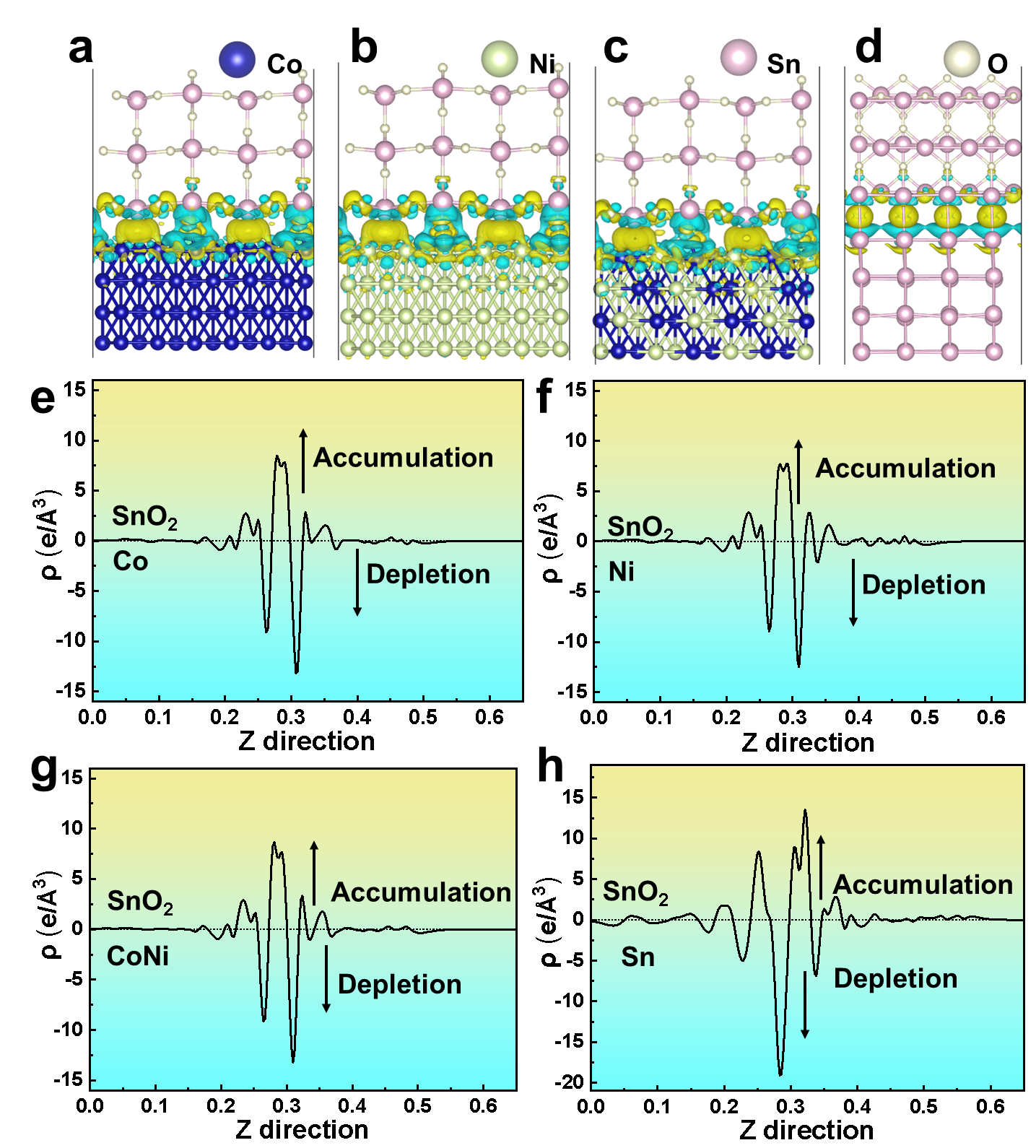


**Fig. S16** The DFT calculated electron density distribution of **a,e** Co/SnO_2_ interface, **b,f** Ni/SnO_2_ interface, **c,g** CoNi/SnO_2_ interface, and **d,h** Sn/SnO_2_ interface (the cyan and yellow regions refer to electron depletion and accumulation, respectively)


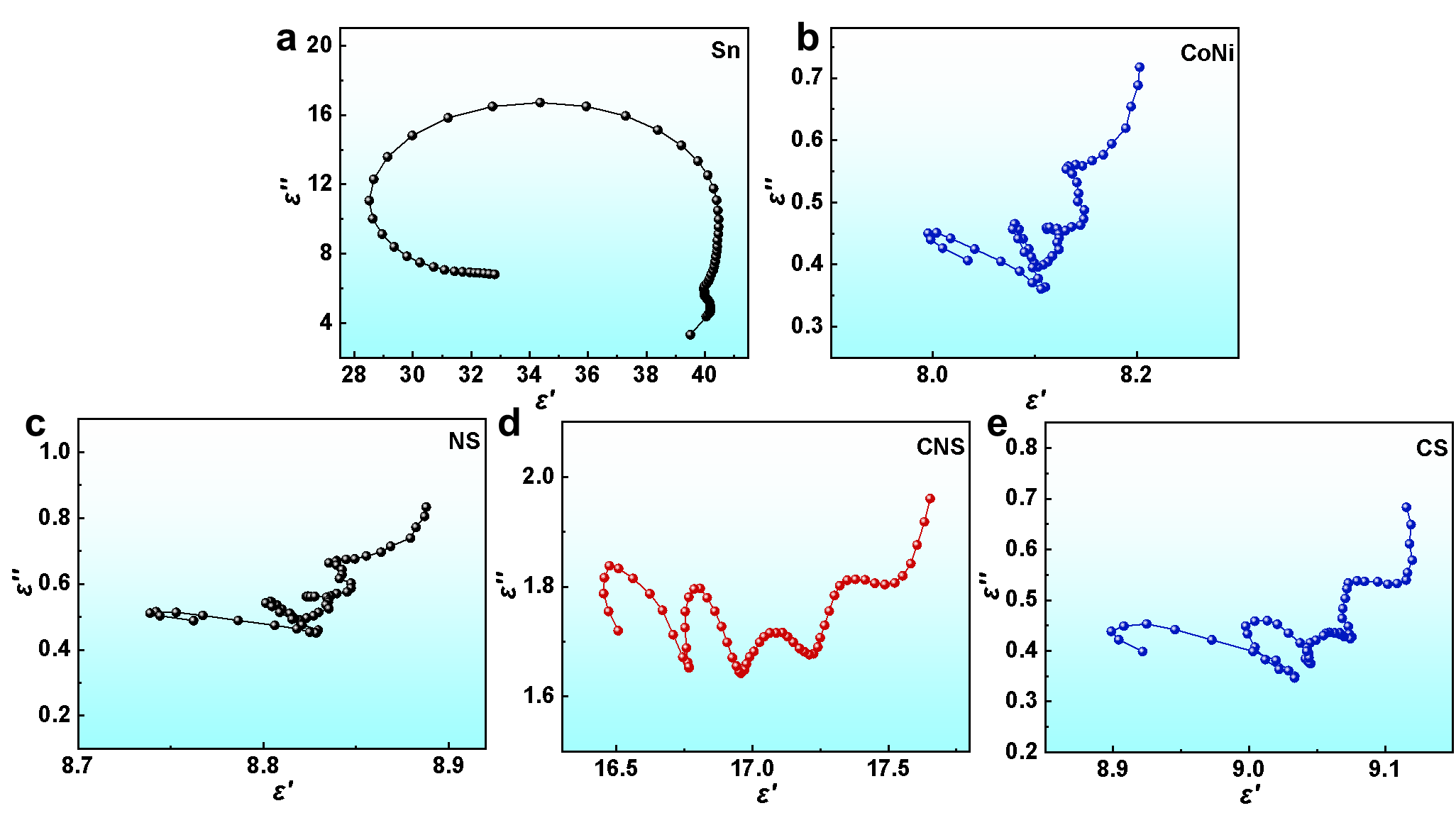


**Fig. S17** Cole-Cole semicircles of **a** Sn, **b** CoNi, **c** NS, **d** CNS, and **e** CS at low frequency


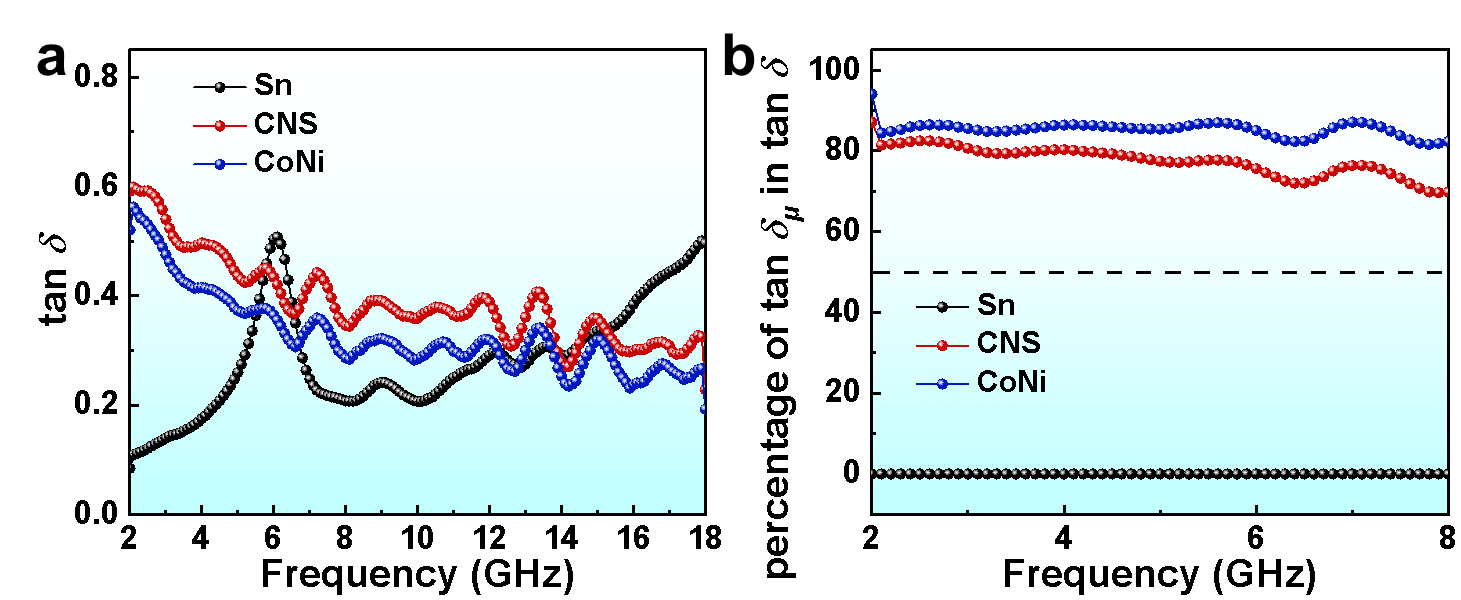


**Fig. S18 a** the total loss tangent (tan δ) and **b** the percentage contribution of the magnetic loss tangent (tan δ_μ_) to tan δ for the samples


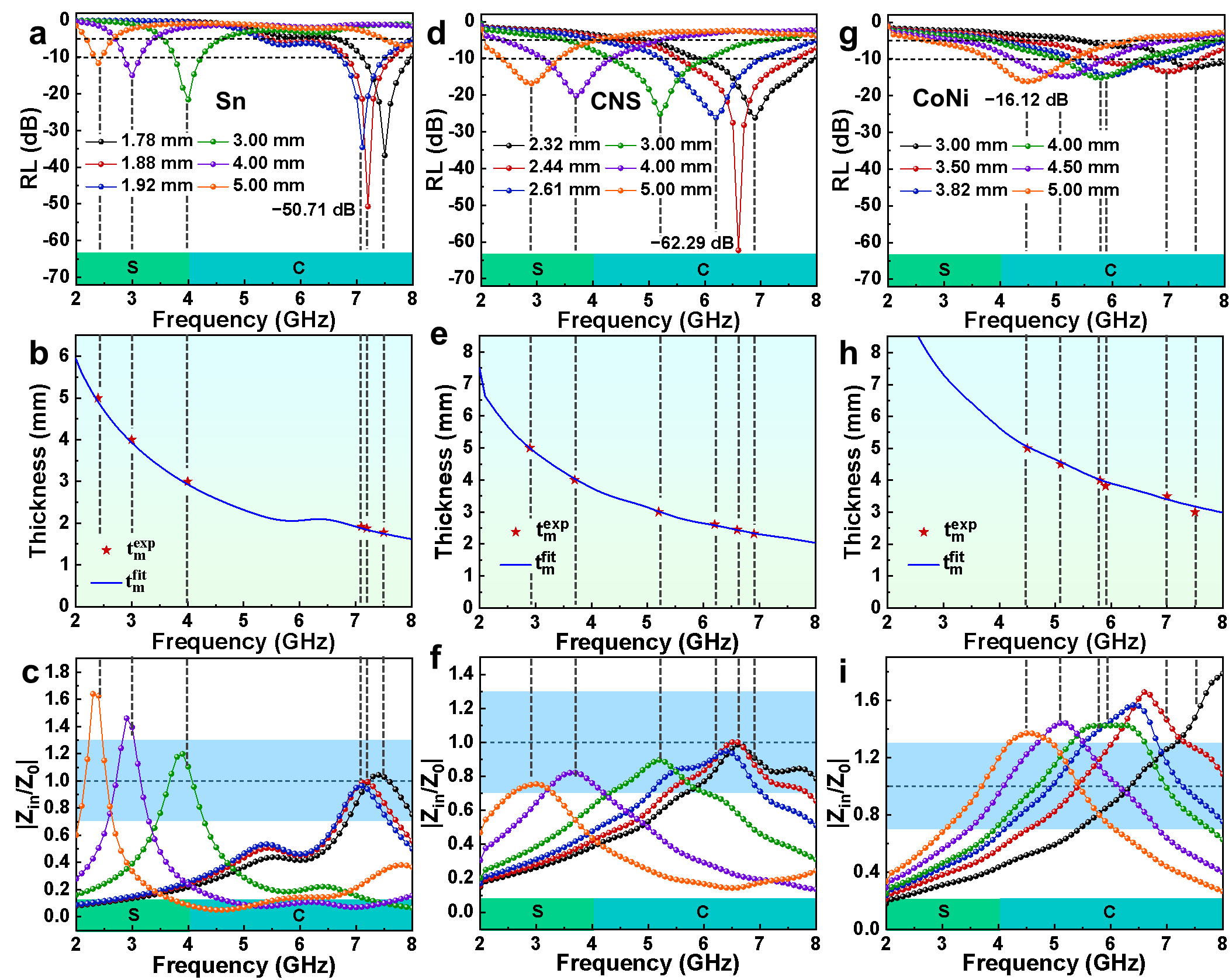


**Fig. S19** 2D plots of **a,d,g** RL, **b,e,h** RL peak frequency corresponds to the experimental value of thickness and the simulated thickness value of the quarter-wavelength model, and **c,f,i** Impedance matching rate (|Z_in_/Z_0_|) of Sn whisker, CNS, and CoNi, respectively


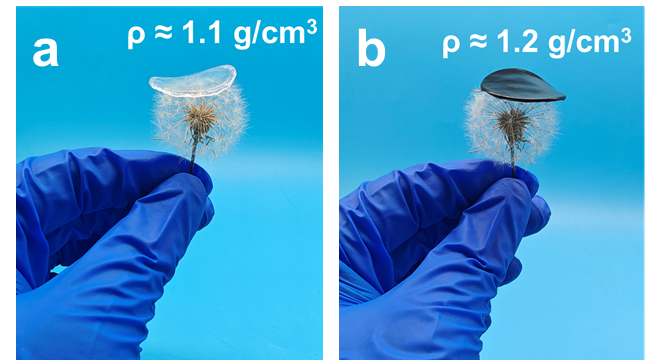


**Fig. S20** Demonstration of low density of **a** TPU and **b** CNS/TPU-2 film


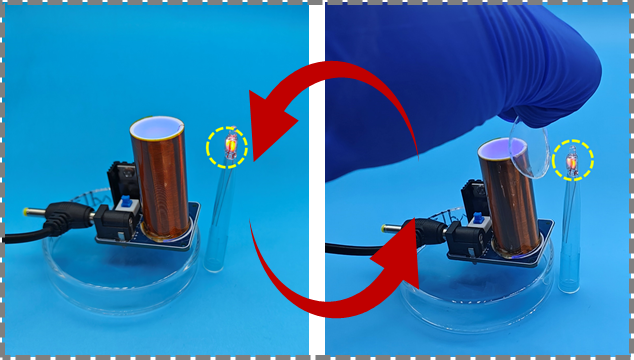


**Fig. S21** Demonstration of the electromagnetic protection (i.e., shielding of electromagnetic signals from the Tesla coil to shut off a light bulb) of the pure TPU film


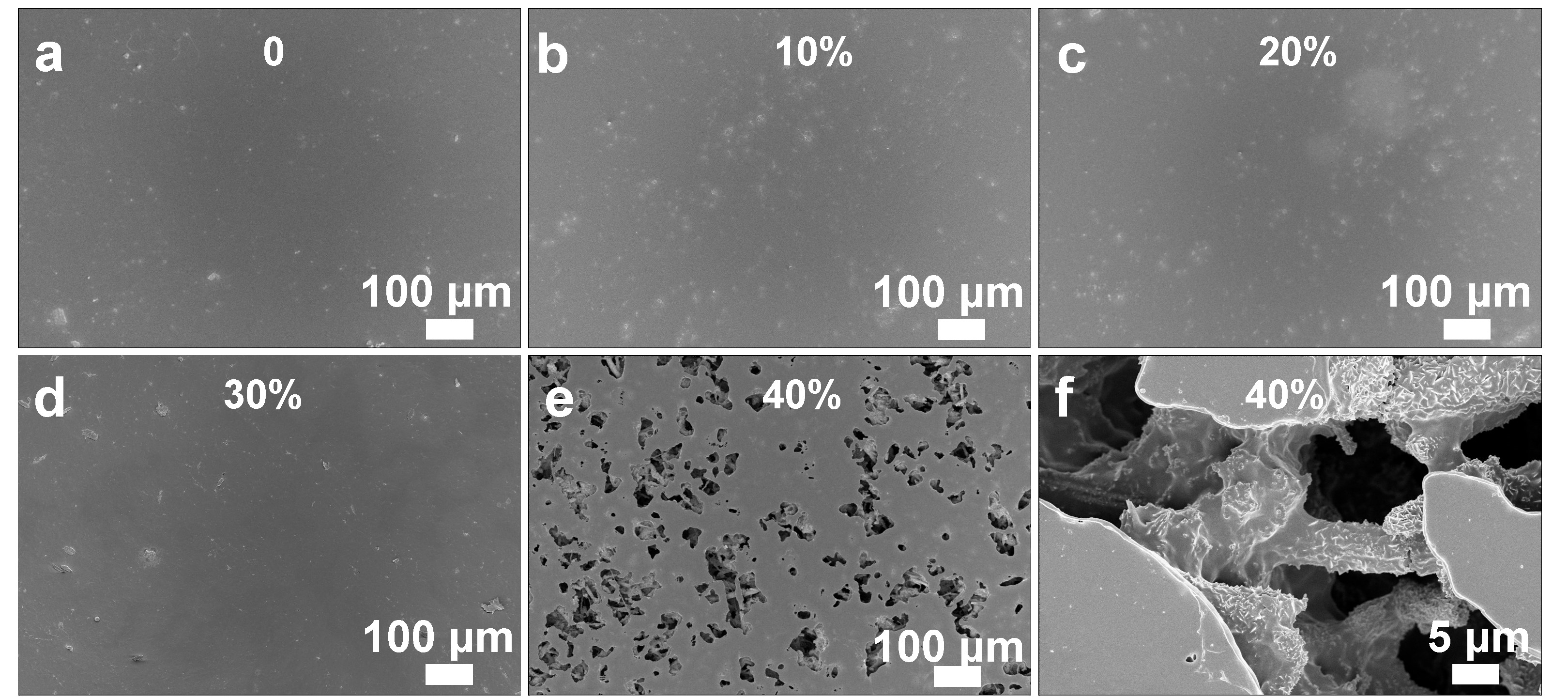


**Fig. S22** SEM images of the surface of TPU films with different filling amount (wt.%) of CNS: **a** 0, **b** 10%, **c** 20%, **d** 30%, and **e,f** 40%


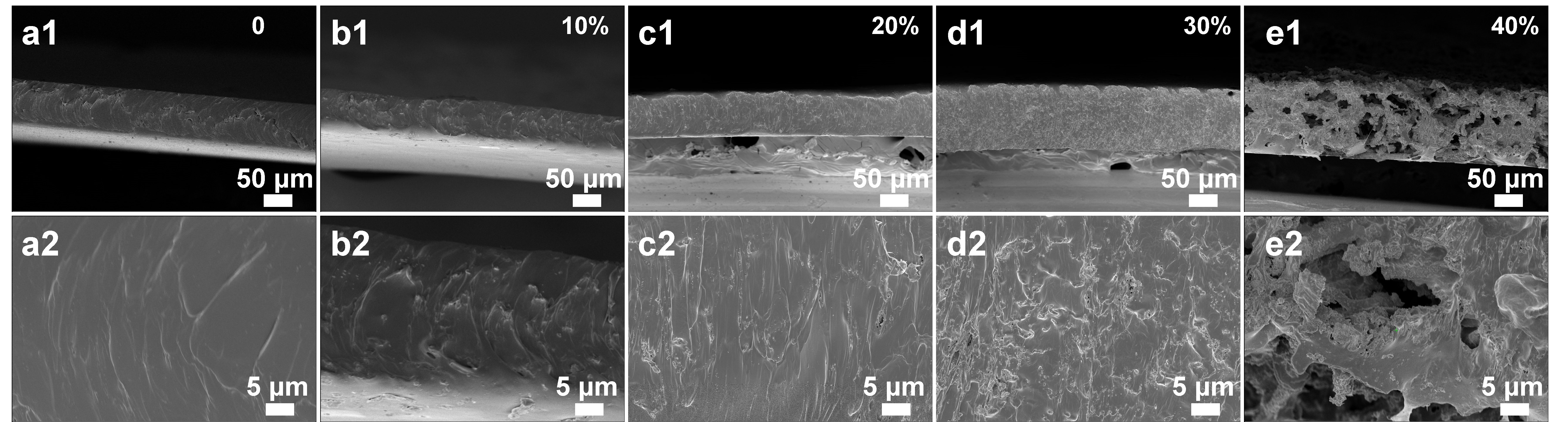


**Fig. S23** SEM images of the cross-section of TPU films with different filling amount (wt.%) of CNS: **a** 0, **b** 10%, **c** 20%, **d** 30%, and **e** 40%


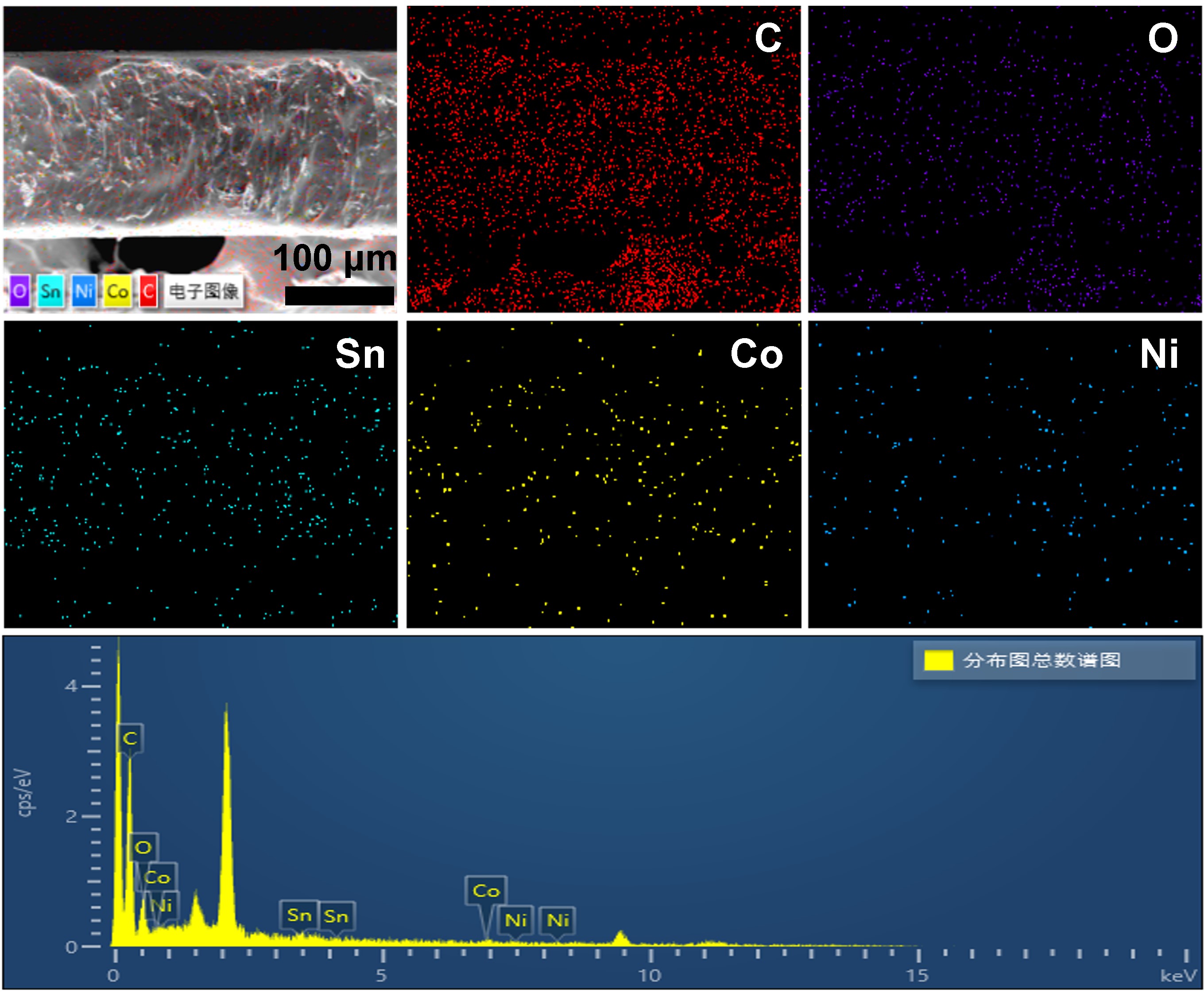


**Fig. S24** EDS mapping of the cross-section of CNS/TPU-2 film


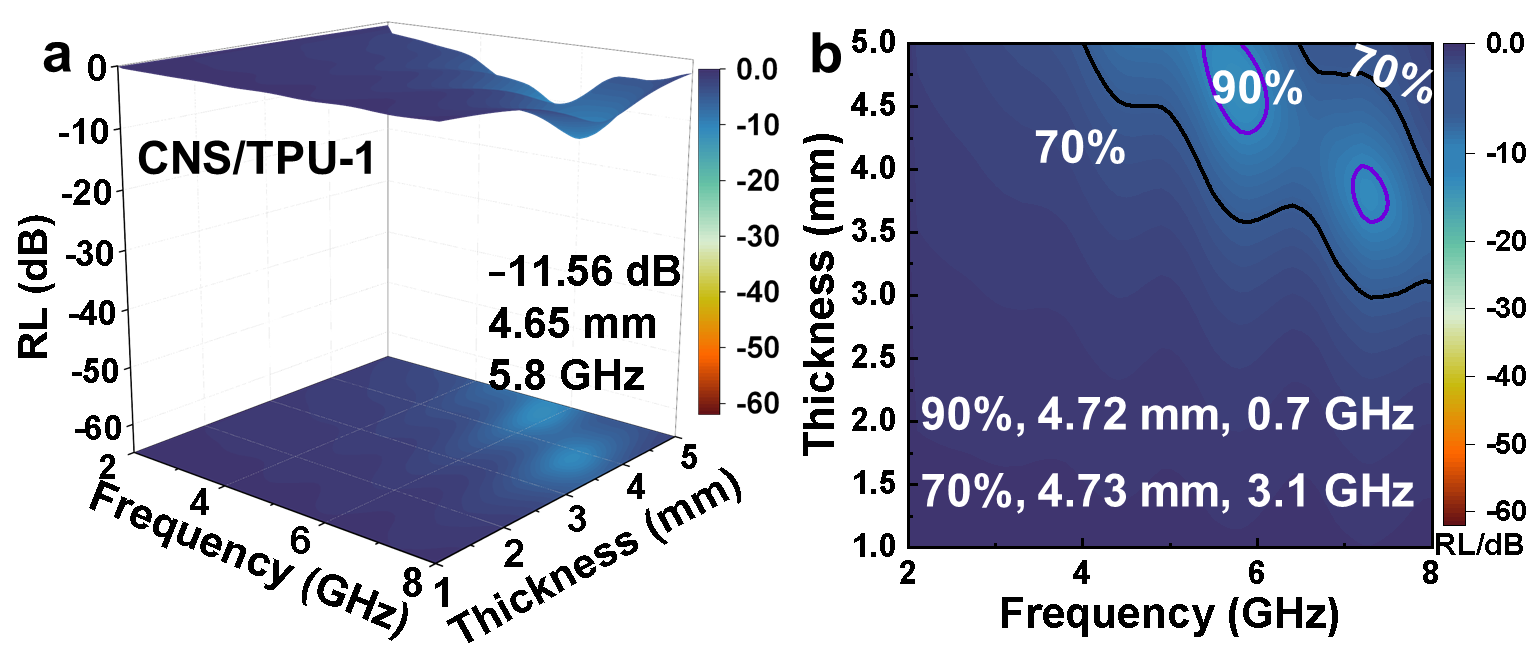


**Fig. S25 a** The RL value and **b** EAB of CNS/TPU-1 film at low-frequency


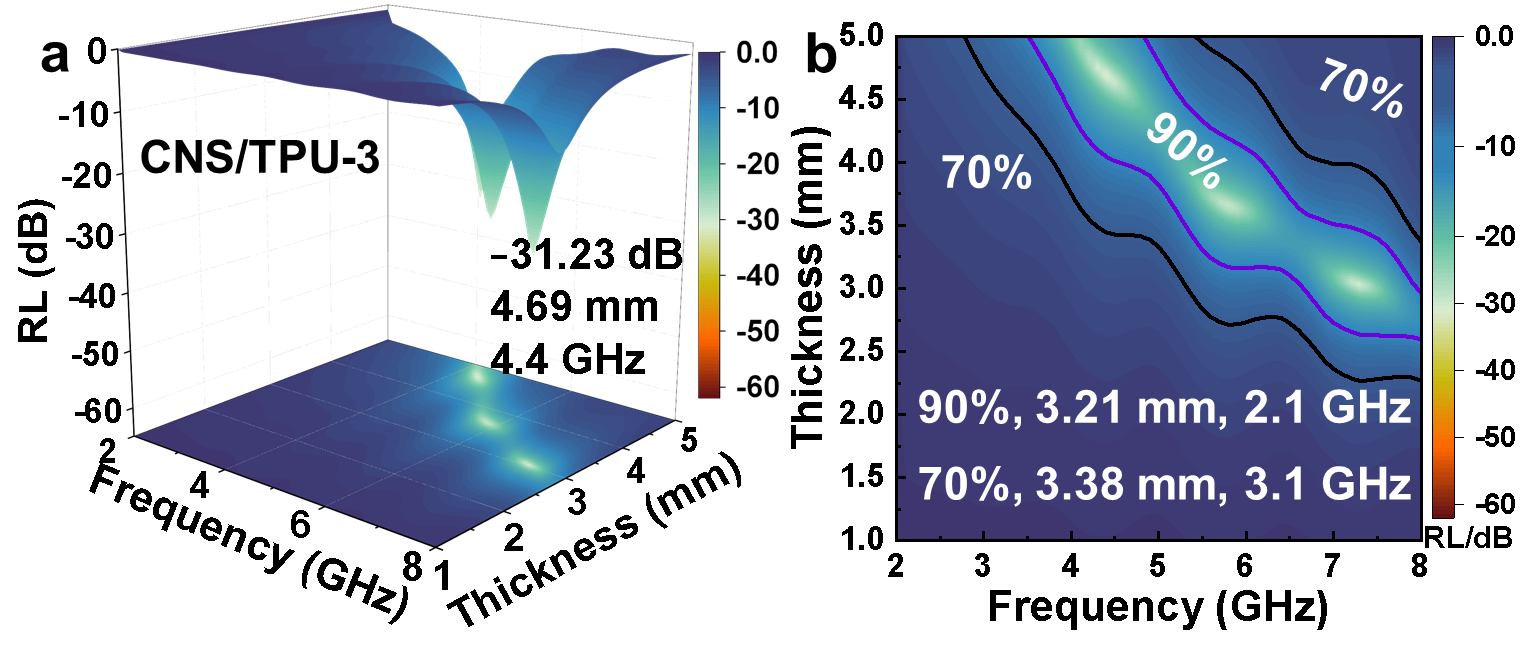


**Fig. S26 a** The RL value and **b** EAB of CNS/TPU-3 film at low-frequency


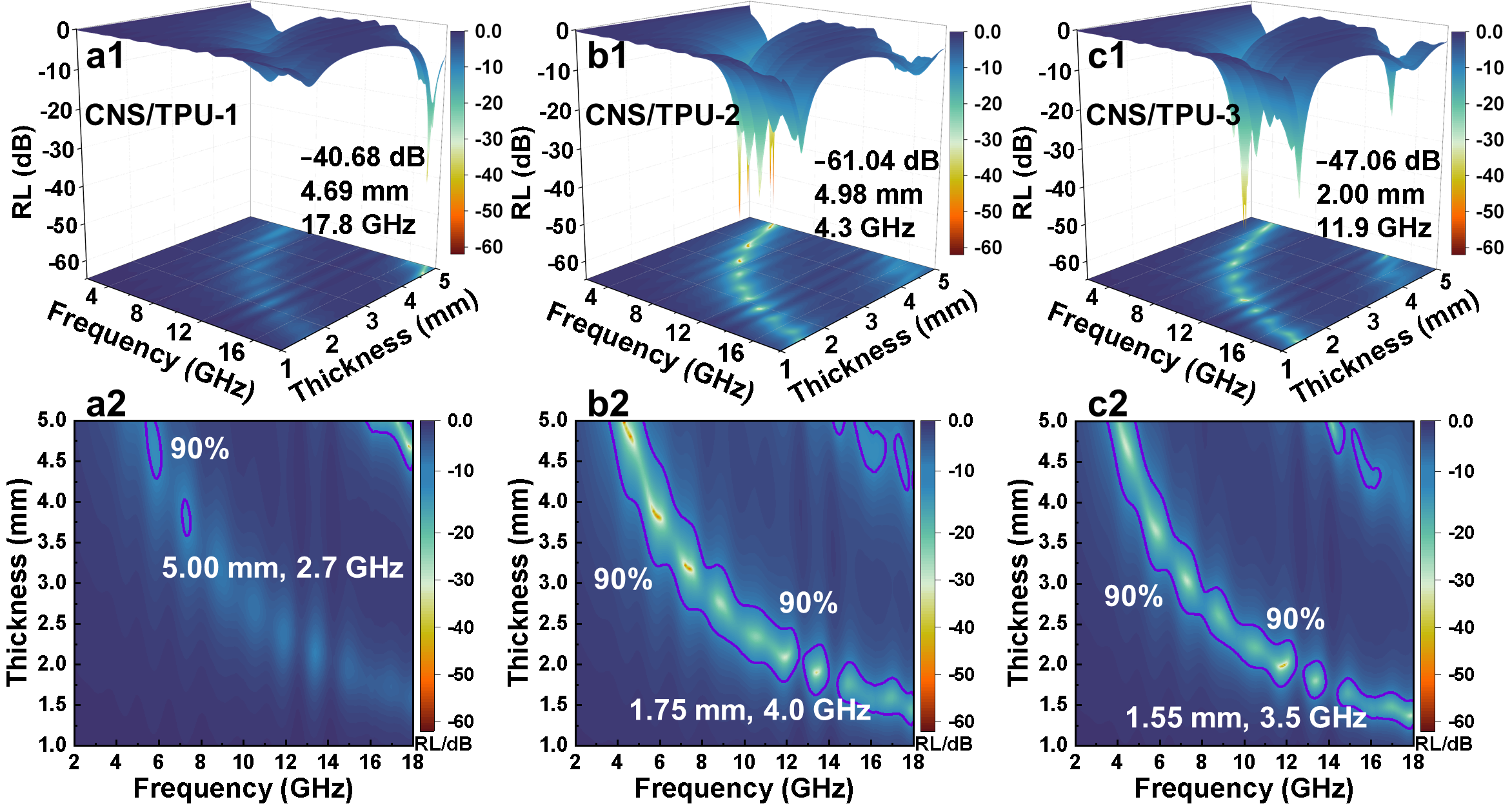


**Fig. S27** The RL value and EAB of **a** CNS/TPU-1, **b** CNS/TPU-2 and **c** CNS/TPU-3 at 2-18 GHz


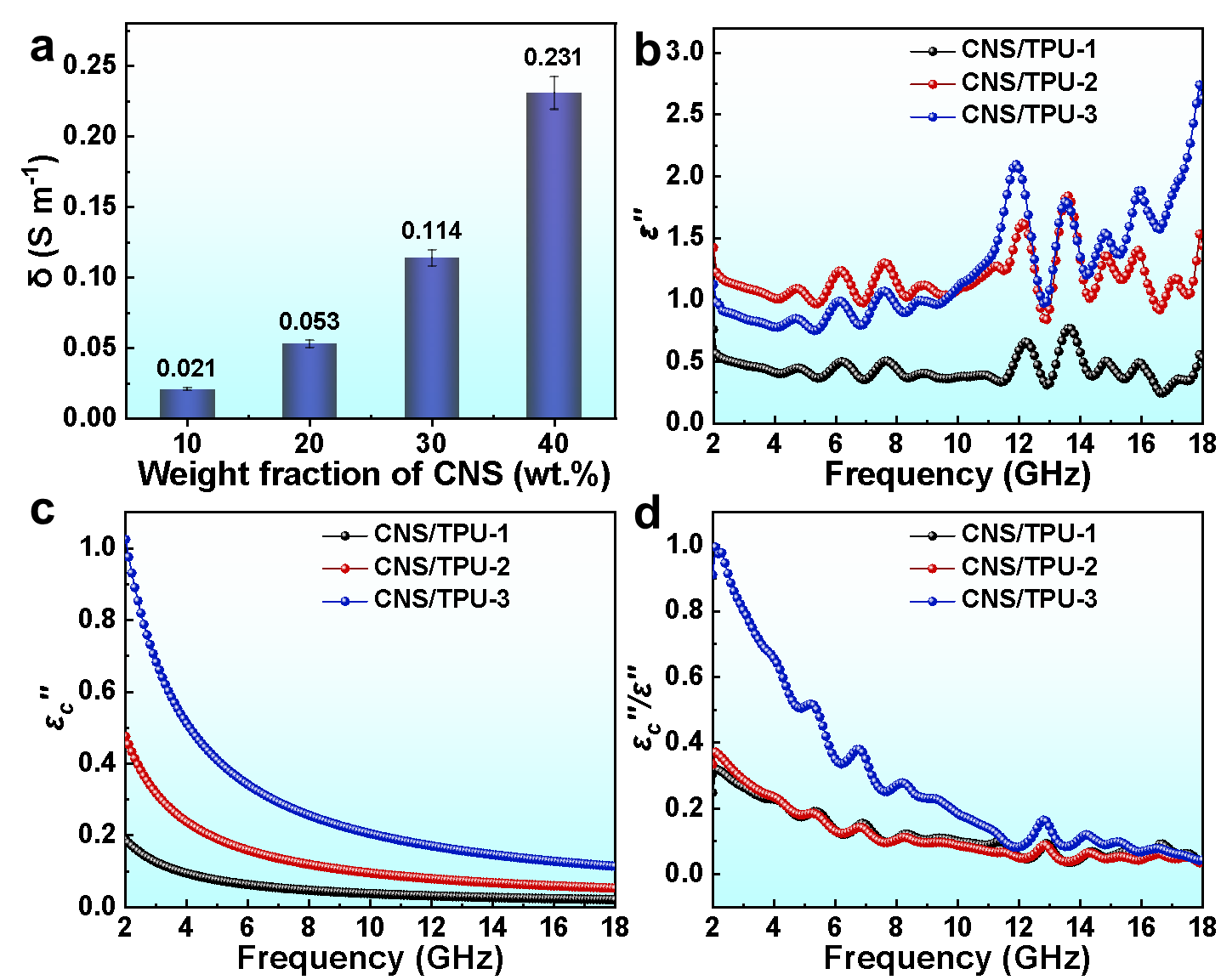


**Fig. S28** **a** DC electrical conductivity of composite films as a function of CNS loading. **b** Imaginary part of the complex permittivity (ε''), **c** Conductive-loss component (ε_c_'') extracted from the dielectric response, and **d** Percentage contribution of conductive loss to the total dielectric loss (ε_c_''/ε'') for CNS/TPU film samples
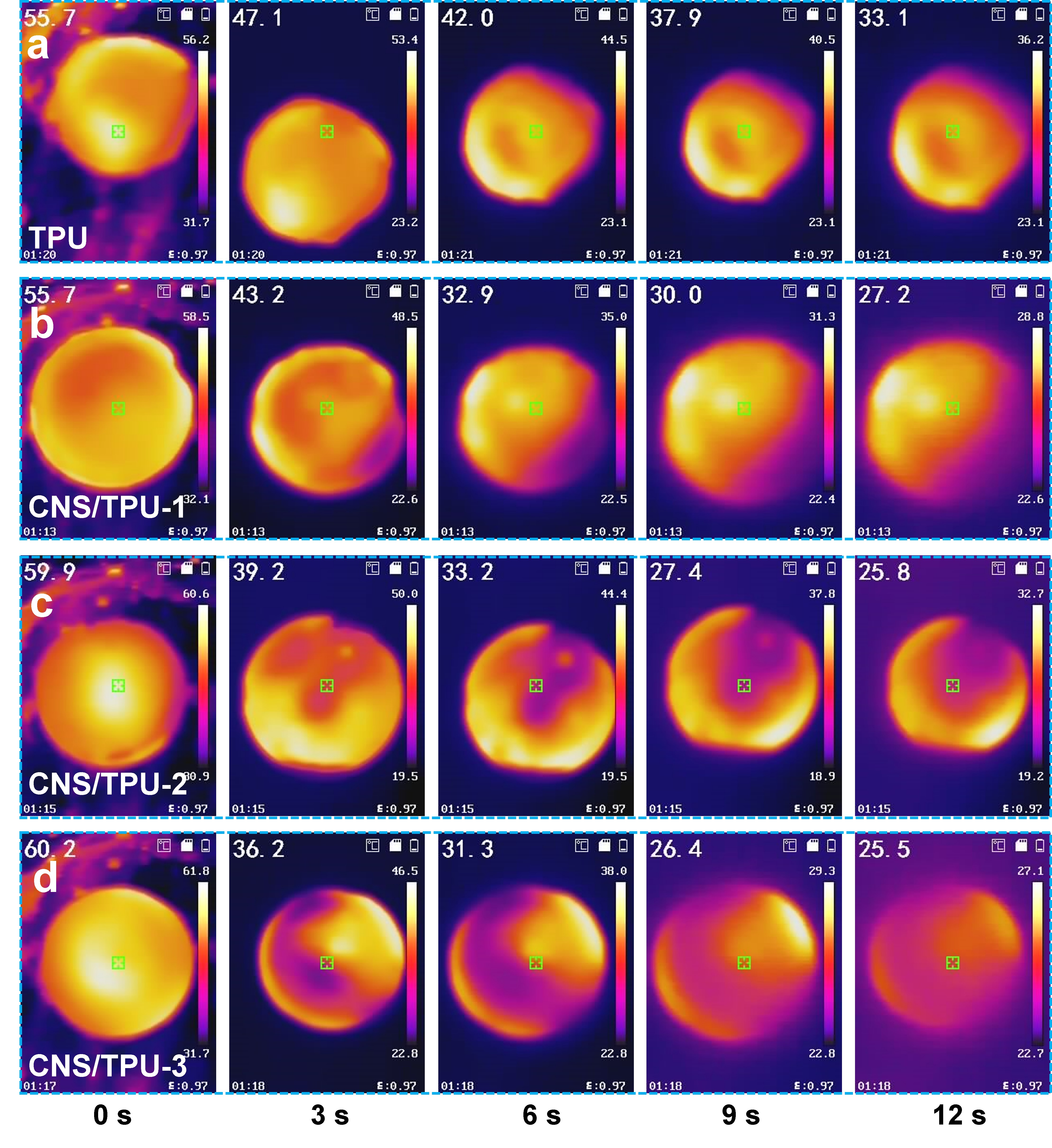


**Fig. S29** Infrared thermal images of TPU films with different CNS fillings dissipating over time at room temperature after leaving a 60 °C hot plate


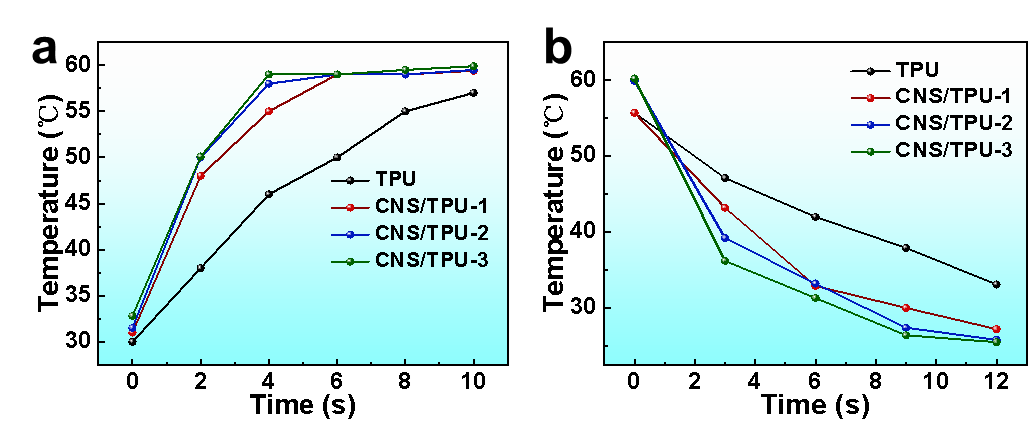


**Fig. S30** **a** Heating temperature-time curves and **b** Cooling temperature-time curves of TPU films with different CNS fillings

**Table S1** Low-frequency microwave absorption properties of absorbers

| Materials | RL_min_ (dB) | \|RL_min_\|/d (dB/mm) | EAB (GHz) | EAB/d (GHz/mm) | Refs. |
| --- | --- | --- | --- | --- | --- |
| NiFe_2_O_4_ @BiFeO_3_@PPy | -65.30 | 14.74 | 2.32 | 0.52 | [S1] |
| FeCo | -42.1 | 19.14 | 1.6 | 0.73 | [S2] |
| CoNi@BN/PDMS | -49.9 | 11.34 | 2.40 | 0.16 | [S3] |
| Fe_6_W_6_C-FeWO_4_@C | -36.5 | 8.11 | 2.6 | 0.79 | [S4] |
| FeCo@C | -35.90 | 8.98 | 2.58 | 0.65 | [S5] |
| CoNi/PDMS | -56.7 | 13.83 | 1.04 | 0.25 | [S6] |
| 3D MXene/CNF | -47.9 | 5.64 | 1.2 | 0.14 | [S7] |
| **CNS** | **-62.29** | **25.53** | **2.2** | **0.95** | **This work** |
| **CNS/TPU-2** | **-61.04** | **12.26** | **2.5** | **0.75** | **This work** |

**Table S2** M_s_ and H_c_ of samples

| Materials | CS | NS | CoNi | **CNS** |
| --- | --- | --- | --- | --- |
| **Ms (emu/g)** | 43.81 | 30.19 | 108.53 | **44.64** |
| **Hc (oe)** | 135.89 | 116.57 | 99.73 | **160.27** |

**Table S3** |K_1_| of samples

| Materials | CS | NS | **CNS** |
| --- | --- | --- | --- |
| \|K_1_\| (J/kg) | 0.298 | 0.176 | **0.358** |

# Supplementary References

1. B. Cai, L. Zhou, P.-Y. Zhao, H.-L. Peng, Z.-L. Hou et al., Interface-induced dual-pinning mechanism enhances low-frequency electromagnetic wave loss. Nat Commun. **15**, 3299 (2024). <https://doi.org/10.1038/s41467-024-47537-5>
2. G. Chen, R. Zhang, M. Yuan, S. Xue, Y. Liu et al., Visualizing nanoscale interlayer magnetic interactions and unconventional low‐frequency behaviors in ferromagnetic multi‐shelled structures. Adv. Mater. **36**, 2313411 (2024). <https://doi.org/10.1002/adma.202313411>
3. M. He, X. Zhong, X. Lu, J. Hu, K. Ruan et al., Excellent low-frequency microwave absorption and high thermal conductivity in polydimethylsiloxane composites endowed by hydrangea-like coni@bn heterostructure fillers. Adv. Mater. **36**, 2410186 (2024). <https://doi.org/10.1002/adma.202410186>
4. L. Rao, M. Huang, X. Wang, Y. Qian, Z. Yan et al., Atomic infusion induced reconstruction enhances multifunctional thermally conductive films for robust low-frequency electromagnetic absorption. Angew. Chem. Int. Ed. **64**, e202418338 (2025). <https://doi.org/10.1002/anie.202418338>
5. L. Rao, L. Wang, C. Yang, R. Zhang, J. Zhang et al., Confined diffusion strategy for customizing magnetic coupling spaces to enhance low‐frequency electromagnetic wave absorption. Adv. Funct. Mater. **33**, 2213258 (2023). <https://doi.org/10.1002/adfm.202213258>
6. M. He, J. Hu, H. Yan, X. Zhong, Y. Zhang et al., Shape anisotropic chain‐like coni/polydimethylsiloxane composite films with excellent low‐frequency microwave absorption and high thermal conductivity. Adv Funct Mater. 2316691 (2024). <https://doi.org/10.1002/adfm.202316691>
7. B. Shan, Y. Wang, X. Ji, Y. Huang, Enhancing low-frequency microwave absorption through structural polarization modulation of mxenes. Nano-Micro Lett. **16**, 212 (2024). <https://doi.org/10.1007/s40820-024-01437-x>
